# Relativistic Rational Extended Thermodynamics of Polyatomic Gases with a New Hierarchy of Moments

**DOI:** 10.3390/e24010043

**Published:** 2021-12-26

**Authors:** Takashi Arima, Maria Cristina Carrisi, Sebastiano Pennisi, Tommaso Ruggeri

**Affiliations:** 1 Department of Engineering for Innovation, National Institute of Technology, Tomakomai College, Tomakomai 059-1275, Japan; arima@tomakomai-ct.ac.jp; 2 Department of di Mathematics and Informatics, University of Cagliari, 09124 Cagliari, Italy; mariacri.carrisi@unica.it (M.C.C.); spennisi@unica.it (S.P.); 3 Department of Mathematics and Alma Mater Research Center on Applied Mathematics AM^2^, University of Bologna, 40126 Bologna, Italy

**Keywords:** relativistic extended thermodynamics, rarefied polyatomic gas, causal theory of relativistic fluids

## Abstract

A relativistic version of the rational extended thermodynamics of polyatomic gases based on a new hierarchy of moments that takes into account the total energy composed by the rest energy and the energy of the molecular internal mode is proposed. The moment equations associated with the Boltzmann–Chernikov equation are derived, and the system for the first 15 equations is closed by the procedure of the maximum entropy principle and by using an appropriate BGK model for the collisional term. The entropy principle with a convex entropy density is proved in a neighborhood of equilibrium state, and, as a consequence, the system is symmetric hyperbolic and the Cauchy problem is well-posed. The ultra-relativistic and classical limits are also studied. The theories with 14 and 6 moments are deduced as principal subsystems. Particularly interesting is the subsystem with 6 fields in which the dissipation is only due to the dynamical pressure. This simplified model can be very useful when bulk viscosity is dominant and might be important in cosmological problems. Using the Maxwellian iteration, we obtain the parabolic limit, and the heat conductivity, shear viscosity, and bulk viscosity are deduced and plotted.

## 1. Introduction

Rational extended thermodynamics (RET) is a theory applicable to nonequilibrium phenomena out of local equilibrium. It is expressed by a hyperbolic system of field equations with local constitutive equations and is strictly related to the kinetic theory with the closure method of the hierarchies of moment equations in both classical and relativistic frameworks [1,2].

The first relativistic version of the modern RET was given by Liu, Müller, and Ruggeri (LMR) [3] considering the Boltzmann–Chernikov relativistic equation [4,5,6]:

(1) $$p^{\alpha }\partial_{\alpha }f=Q,$$

in which the distribution function *f* depends on $\left(x^{\alpha },p^{\beta }\right)$, where $x^{\alpha }$ are the space-time coordinates, $p^{\alpha }$ is the four-momentum, $\partial_{\alpha }=\partial /\partial x^{\alpha }$, *Q* is the collisional term, and α,β=0,1,2,3. For monatomic gases, the relativistic moment equations associated with (1), truncated at tensorial index N+1 are:

(2) $$\partial_{\alpha }A^{\alpha \alpha_{1}\cdots \alpha_{n}}=I^{\alpha_{1}\cdots \alpha_{n}}\;with\;n=0\;,\;\cdots \;,\;N,$$

with

(3) $$\begin{array}{c} A^{\alpha \alpha_{1}\cdots \alpha_{n}}=\frac{c}{m^{n-1}}\int_{R^{3}}f\;p^{\alpha }p^{\alpha_{1}}\cdots p^{\alpha_{n}}\;\;dP,\;I^{\alpha_{1}\cdots \alpha_{n}}=\frac{c}{m^{n-1}}\int_{R^{3}}Q\;p^{\alpha_{1}}\cdots p^{\alpha_{n}}\;\;dP, \end{array}$$

where *c* denotes the light velocity, *m* is the particle mass in the rest frame, and

$$dP=\frac{dp^{1}\;dp^{2}\;dp^{3}}{p^{0}}.$$

If n=0, the tensor reduces to $A^{\alpha }$; moreover, the production tensor in the right-side of (2) is zero for n=0,1, because the first 5 equations represent the conservation laws of the particle number and of the energy-momentum, respectively.

When N=1, we have the relativistic Euler system

(4) $$\partial_{\alpha }A^{\alpha }=0,\;\partial_{\alpha }A^{\alpha \beta }=0,$$

where, also in the following, $A^{\alpha }\equiv V^{\alpha }$ and $A^{\alpha \beta }\equiv T^{\alpha \beta }$ have the physical meaning, respectively, of the particle number vector and the energy-momentum tensor. Instead, when N=2, we have the LMR theory of a relativistic gas with 14 fields:

(5) $$\partial_{\alpha }A^{\alpha }=0,\;\partial_{\alpha }A^{\alpha \beta }=0,\;\partial_{\alpha }A^{\alpha \beta \gamma }=I^{\beta \gamma },\;\left(\gamma =0,1,2,3;\;\;I_{\;\;\alpha }^{\alpha }=0\right).$$

Recently, Pennisi and Ruggeri first constructed a relativistic RET theory for polyatomic gases with (2) in the case of N=2 [7] (see also [8,9]) whose moments are given by

(6) $$\begin{array}{c} \begin{array}{cc} \; & A^{\alpha }=mc\int_{R^{3}}\int_{0}^{\infty }fp^{\alpha }\phi \left(I\right)\;dI\;dP\;,\\ \; & A^{\alpha \beta }=\frac{1}{mc}\int_{R^{3}}\int_{0}^{\infty }fp^{\alpha }p^{\beta }\left(mc^{2}+I\right)\;\phi \left(I\right)\;dI\;dP\;,\\ \; & A^{\alpha \beta \gamma }=\frac{1}{m^{2}c}\int_{R^{3}}\int_{0}^{+\infty }f\;p^{\alpha }p^{\beta }p^{\gamma }\;\left(mc^{2}+2I\right)\;\phi \left(I\right)\;dI\;dP\;, \end{array} \end{array}$$

where the distribution function $f(x^{\alpha },p^{\beta },I)$ depends on the extra variable I, similar to the classical one (see [2] and references therein), that has the physical meaning of the *molecular internal energy of internal modes* in order to take into account the exchange of energy due to the rotation and vibration of a molecule, and ϕ(I) is the state density of the internal mode.

In [7], by taking the traceless part of the third order tensor (i.e., $A^{\alpha \langle \beta \gamma \rangle }$) as a field instead of $A^{\alpha \beta \gamma }$ in (5)${}_{3}$, the relativistic theory with 14 fields (RET${}_{14}$) was proposed. It was also shown that its classical limit coincides with the classical RET${}_{14}$ based on the binary hierarchy [2,10,11]. The beauty of the relativistic counterpart is that there exists a single hierarchy of moments, but, as was noticed by the authors, to obtain the classical theory of RET${}_{14}$, it was necessary to put the factor 2 in front of I in the last equation of (6)! This was also more evident in the theory with any number of moments, where Pennisi and Ruggeri generalized (6) considering the following moments [12]:

(7) $$\begin{array}{c} \begin{array}{cc} \; & A^{\alpha \alpha_{1}\cdots \alpha_{n}}=\frac{1}{m^{n}c}\int_{R^{3}}\int_{0}^{+\infty }f\;p^{\alpha }p^{\alpha_{1}}\cdots p^{\alpha_{n}}\;\left(mc^{2}+nI\right)\;\phi \left(I\right)\;dI\;dP\;,\\ \; & I^{\alpha_{1}\cdots \alpha_{n}}=\frac{1}{m^{n}c}\int_{R^{3}}\int_{0}^{+\infty }Q\;p^{\alpha_{1}}\cdots p^{\alpha_{n}}\;\left(mc^{2}+nI\right)\;\phi \left(I\right)\;dI\;dP. \end{array} \end{array}$$

In this case, we need a factor nI in (7) to obtain, in the classical limit, the binary hierarchy.

To avoid this unphysical situation, Pennisi first noticed that $\left(mc^{2}+nI\right)$ appearing in (7) are the first two terms of the Newton binomial formula for ${\left(mc^{2}+I\right)}^{n}/{\left(mc^{2}\right)}^{n-1}$. Therefore he proposed in [13] to modify, in the relativistic case, the definition of the moments by using the substitution:

$${\left(mc^{2}\right)}^{n-1}\left(mc^{2}+nI\right)\;\mathrm{with}\;\;{\left(mc^{2}+I\right)}^{n},$$

that is, instead of (7), the following moments are proposed:

(8) $$\begin{array}{c} \begin{array}{cc} \; & A^{\alpha \alpha_{1}\cdots \alpha_{n}}={\left(\frac{1}{mc}\right)}^{2n-1}\int_{R^{3}}\int_{0}^{+\infty }f\;p^{\alpha }p^{\alpha_{1}}\cdots p^{\alpha_{n}}\;{\left(mc^{2}+I\right)}^{n}\;\phi \left(I\right)\;dI\;dP\;,\\ \; & I^{\alpha_{1}\cdots \alpha_{n}}={\left(\frac{1}{mc}\right)}^{2n-1}\int_{R^{3}}\int_{0}^{+\infty }Q\;p^{\alpha_{1}}\cdots p^{\alpha_{n}}\;{\left(mc^{2}+I\right)}^{n}\;\phi \left(I\right)\;dI\;dP. \end{array} \end{array}$$

Such definitions are more physical because now the full energy (the sum of the rest frame energy and the energy of internal modes) $mc^{2}+I$ appears in the moments.

The aim of this paper is to consider the system (5) with moments given by (8). In this way, for the case with N=2 also, by taking the trace part of $A^{\alpha \beta \gamma }$ as a field, we have 15 field equations, and to close the system, we adopt the molecular procedure of RET based on the maximum entropy principle.

The paper is organized as follows. In Section 2, the values of generic moments in an equilibrium state are estimated in the general case. In Section 3, the RET theory for 15 fields (RET${}_{15}$) is proposed, and the constitutive quantities are closed near the equilibrium state. By adopting a variant of the BGK model appropriate for polyatomic gases proposed by Pennisi and Ruggeri [14], the production tensor is derived. In Section 4, the four-dimensional entropy flux and the entropy production are deduced within the second order with respect to the nonequilibrium variables. Then, we show the condition of convexity of the entropy density and the positivity of the entropy production, which ensure the well-posedness of the Cauchy problem and the entropy principle as a result. We also discuss in Section 5 the case of the diatomic gases for which all coefficients are expressed in closed form in terms of the ratio of two Bessel functions, similar to the case of monatomic gases. In Section 6, we study the ultra-relativistic limit. In Section 7, the principal subsystems of RET${}_{15}$ are studied. First, we obtain RET${}_{14}$ in which all field variables have physical meaning. Then, at the same level as RET${}_{14}$ in the sense of the principal subsystem, there also exists the subsystem with 6 fields in which the dissipation is only due to the dynamical pressure. This system is important in the case that the bulk viscosity is dominant compared to the shear viscosity and heat conductivity, and it must be particularly interesting in cosmological problems. The simplest subsystem is the Euler non-dissipative case with 5 fields. In Section 8, we use the Maxwellian iteration and, as a result, the phenomenological coefficients of the Eckart theory, that is, the heat conductivity, shear viscosity, and bulk viscosity are determined with the present model. Finally, in Section 9, we show that the classic limit of the present model coincides with the classical RET${}_{15}$ studied in [15].

## 2. Distribution Function and Moments at Equilibrium

The equilibrium distribution function $f_{E}$ of polyatomic gas that generalizes the Jüttner one of monatomic gas was evaluated in [7] with the variational procedure of the maximum entropy principle (MEP) [1,16,17,18]. Considering the first 5 balance equations of (5) in equilibrium state:

$$A_{E}^{\alpha }\equiv V_{E}^{\alpha }=m\;nU^{\alpha },\;\;A_{E}^{\alpha \beta }\equiv T_{E}^{\alpha \beta }=ph^{\alpha \beta }+\frac{e}{c^{2}}\;U^{\alpha }U^{\beta }.$$

MEP requires that the appropriate distribution function $f\equiv f(x^{\alpha },p^{\alpha },I)$ is the one which maximizes the entropy density

$$\rho S=h_{E}=h_{E}^{\alpha }U_{\alpha }=-k_{B}\;c\;U_{\alpha }\int_{R^{3}}\int_{0}^{+\infty }f\ln fp^{\alpha }\phi \left(I\right)\;dI\;dP\;,$$

under the constraints that the temporal parts $V^{\alpha }U_{\alpha }$ and $T^{\alpha \beta }U_{\beta }$ are prescribed. Here, $k_{B},n,\rho \left(=nm\right),U^{\alpha },h^{\alpha \beta },p,e,S$ are, respectively, the Boltzmann constant, the particle number, the mass density, the four-velocity ($U^{\alpha }U_{\alpha }=c^{2}$), the projector tensor $\left(h^{\alpha \beta }=U^{\alpha }U^{\beta }/c^{2}-g^{\alpha \beta }\right)$, the pressure, the energy, and the entropy density, and $g^{\alpha \beta }=\mathrm{diag}\left(1\;,\;-1\;,\;-1\;,\;-1\right)$ is the metric tensor.

The equilibrium distribution function for a rarefied polyatomic gas that maximizes the entropy has the following expression [7]:

(9) $$f_{E}=\frac{n}{\bar{A}\left(\gamma \right)}\frac{1}{4\pi m^{3}c^{3}}e^{-\frac{1}{k_{B}T}\left[\left(1+\frac{I}{mc^{2}}\right)U_{\beta }p^{\beta }\right]},\;\bar{A}\left(\gamma \right)=\int_{0}^{+\infty }J_{2,1}^{*}\;\phi \left(I\right)\;d\;I$$

with *T* being the absolute temperature,

$$\begin{array}{cc} \; & J_{m,n}^{*}=J_{m,n}\left(\gamma^{*}\right),\;\gamma^{*}=\gamma \;\left(1+\frac{I}{m\;c^{2}}\right),\;\gamma =\frac{m\;c^{2}}{k_{B}T}, \end{array}$$

and

$$J_{m,n}\left(\gamma \right)=\int_{0}^{+\infty }e^{-\gamma \cosh s}\sinh^{m}s\cosh^{n}s\;d\;s\;,$$

subjected to the following recurrence relations [3,7]:

(10) $$\begin{array}{c} J_{m+2,n}\left(\gamma \right)=J_{m,n+2}\left(\gamma \right)-J_{m,n}\left(\gamma \right)\;, \end{array}$$

(11) $$\begin{array}{c} -\gamma J_{m+2,n}\left(\gamma \right)=nJ_{m,n-1}\left(\gamma \right)-\left(n+m+1\right)J_{m,n+1}\left(\gamma \right)\;. \end{array}$$

The pressure and the energy compatible with the equilibrium distribution function (9) are [7]:

(12) $$\begin{array}{c} \begin{array}{cc} \; & p=\frac{k_{B}}{m}\;\rho T\;,\;\;e=\rho c^{2}\omega \left(\gamma \right),\\ \; & \mathrm{with}\;\;\omega \left(\gamma \right)=\frac{\int_{0}^{+\infty }J_{2,2}^{*}\;\left(1+\frac{I}{mc^{2}}\right)\;\phi \left(I\right)\;d\;I}{\int_{0}^{+\infty }J_{2,1}^{*}\;\phi \left(I\right)\;d\;I}. \end{array} \end{array}$$

Taking into account that $e=\rho c^{2}+\rho \varepsilon ,$ where ε is the internal energy, we deduce from (12):

(13) $$\varepsilon =c^{2}\left(\omega -1\right).$$

Therefore, the internal energy is a function only of γ or, it is the same, of *T* as in the classical case for rarefied gases.

The moments in equilibrium state $A_{E}^{\alpha \alpha_{1}\cdots \alpha_{j}}$ for j≥2 were deduced in [13]:

(14) $$\begin{array}{c} A_{E}^{\alpha_{1}\cdots \alpha_{j+1}}=\sum_{k=0}^{\left[\frac{j+1}{2}\right]}\rho c^{2k}\theta_{k,j}\;h^{(\alpha_{1}\alpha_{2}}\cdots h^{\alpha_{2k-1}\alpha_{2k}}U^{\alpha_{2k+1}}\cdots U^{\alpha_{j+1})}\;, \end{array}$$

where

(15) $$\begin{array}{c} \theta_{k,j}=\frac{1}{2k+1}\left(\begin{array}{c} j+1\\ 2k \end{array}\right)\frac{\int_{0}^{+\infty }J_{2k+2,j+1-2k}^{*}\;{\left(1+\frac{I}{mc^{2}}\right)}^{j}\;\phi \left(I\right)\;d\;I}{\int_{0}^{+\infty }J_{2,1}^{*}\;\phi \left(I\right)\;d\;I}\; \end{array}$$

are dimensionless functions depending only on γ. Taking into account (12) and (15), we obtain $\theta_{0,0}=1,\;\theta_{0,1}=\omega \left(\gamma \right)$, and using the recurrence Formula (10) and (11), in [13], the following recurrence relations hold:

(16) $$\begin{array}{c} \begin{array}{cc} \; & \theta_{0,0}=1\;,\\ \; & \theta_{0,j+1}=\omega \left(\gamma \right)\;\theta_{0,j}\;-\;\theta_{0,j}^{\;\prime }\;with{\;}^{\prime }=\frac{d}{d\gamma },\\ \; & \\ \; & \theta_{h,j+1}=\frac{j+2}{\gamma }\left(\theta_{h,j}+\frac{j+3-2h}{2h}\theta_{h-1,j}\right)\;forh=1,\;\cdots ,\;\left[\frac{j+1}{2}\right]\;,\\ \; & \\ \; & \theta_{\frac{j+2}{2},j+1}=\frac{1}{\gamma }\theta_{\frac{j}{2},\;j}\;for\;j\;even\;. \end{array} \end{array}$$

It is interesting to see that all the scalar coefficients can be expressed in terms of the function ω(γ) and of its derivatives with respect to γ (or with respect to the temperature *T*), and ω is strictly related to the internal energy ε by (13). A similar situation is studied in the article [15] for the non-relativistic case.

The values of $\theta_{h,j}$ can be determined, by using the recurrence Formula (16), according to the following diagram:

$$\begin{array}{c} \begin{array}{cccccccccc} \theta_{0,0} & \Rightarrow & \theta_{0,1} & \Rightarrow & \theta_{0,2} & \Rightarrow & \theta_{0,3} & \cdots \\ \; & ↘ & \; & & \\ \; & \; & \theta_{1,1} & \to & \theta_{1,2} & \to & \theta_{1,3} & \cdots & \\ \; & \; & & \; & & ↘\\ \; & \; & \; & \; & \; & \; & \theta_{2,3} & \to & \theta_{2,4} & \cdots \\ \; \end{array} \end{array}$$

We see that all the $\theta_{0,j}$ can be obtained from $\theta_{0,0}$ by using Equation ${\left(16\right)}_{2}$, and the other $\theta_{h,j}$ with j≥h can be obtained from Equations ${\left(16\right)}_{3,4}$. In particular, we can evaluate the following ones that need to be known for the model with 15 fields in the subsequent sections:

(17) $$\begin{array}{c} \begin{array}{cc} \; & \theta_{0,0}=1,\;\theta_{0,1}=\omega ,\;\theta_{0,2}=\omega^{2}-\omega^{\prime },\\ \; & \theta_{0,3}=\omega^{3}+\omega^{\prime \prime }-3\omega \omega^{\prime },\;\theta_{0,4}=\omega^{4}-\omega {}^{\prime \prime \prime }+4\omega \omega^{\prime \prime }+3\omega^{\prime 2}-6\omega^{2}\omega^{\prime },\\ \; & \theta_{1,1}=\frac{1}{\gamma },\;\theta_{1,2}=\frac{3}{\gamma^{2}}\left(\gamma \omega +1\right),\;\theta_{1,3}=\frac{6}{\gamma^{3}}\left[\gamma^{2}\left(\omega^{2}-\omega^{\prime }\right)+2\left(\gamma \omega +1\right)\right],\\ \; & \theta_{1,4}=\frac{10}{\gamma^{4}}\left\{3\gamma \left[\omega \left(\gamma \omega +2\right)-\gamma \omega^{\prime }\right]+6+\gamma^{3}\left(\omega^{3}+\omega^{\prime \prime }-3\omega \omega^{\prime }\right)\right\},\\ \; & \theta_{2,3}=\frac{3}{\gamma^{3}}\left(\gamma \omega +1\right),\;\theta_{2,4}=\frac{15}{\gamma^{4}}\left[\gamma^{2}\left(\omega^{2}-\omega^{\prime }\right)+3\left(\gamma \omega +1\right)\right]. \end{array} \end{array}$$

## 3. The Closure for the 15 Moments Model

In this section, we consider the simplest and physical case, that is, the system (2) for n=0,1,2 with the moments given by (8):

(18) $$\partial_{\alpha }V^{\alpha }=0,\;\partial_{\alpha }T^{\alpha \beta }=0,\;\partial_{\alpha }A^{\alpha \beta \gamma }=I^{\beta \gamma },\;\left(\beta ,\gamma =0,1,2,3\right).$$

with

(19) $$\begin{array}{c} \begin{array}{cc} \; & V^{\alpha }=mc\int_{R^{3}}\int_{0}^{+\infty }f\;p^{\alpha }\;\phi \left(I\right)\;dI\;dP\;,\;T^{\alpha \beta }=c\int_{R^{3}}\int_{0}^{+\infty }f\;p^{\alpha }p^{\beta }\;\left(1+\frac{I}{mc^{2}}\right)\;\phi \left(I\right)\;dI\;dP\;,\\ \; & A^{\alpha \beta \gamma }=\frac{c}{m}\int_{R^{3}}\int_{0}^{+\infty }f\;p^{\alpha }p^{\beta }p^{\gamma }\;{\left(1+\frac{I}{mc^{2}}\right)}^{2}\;\phi \left(I\right)\;dI\;dP\;,\\ \; & I^{\beta \gamma }=\frac{c}{m}\int_{R^{3}}\int_{0}^{+\infty }Q\;p^{\beta }p^{\gamma }\;{\left(1+\frac{I}{mc^{2}}\right)}^{2}\;\phi \left(I\right)\;dI\;dP. \end{array} \end{array}$$

To close the system (19), we adopt the MEP, which requires finding the distribution function that maximizes the non-equilibrium entropy density:

(20) $$h=h^{\alpha }U_{\alpha }=-k_{B}\;c\;U_{\alpha }\int_{R^{3}}\int_{0}^{+\infty }f\ln fp^{\alpha }\phi \left(I\right)\;dI\;dP\;\to \;\max$$

under the constraints that the temporal part $V^{\alpha }U_{\alpha },T^{\alpha \beta }U_{\alpha }$ and $A^{\alpha \beta \gamma }U_{\alpha }$ are prescribed. Proceeding in the usual way as indicated in previous papers of RET (see [2,7]), we obtain:

(21) $$f_{15}=e^{-1-\frac{\chi }{k_{B}}}\;,\;with\;\chi =m\;\lambda \;+\;\lambda_{\mu }\;p^{\mu }\;\left(1+\;\frac{I}{m\;c^{2}}\right)\;+\;\frac{1}{m}\;\lambda_{\mu \nu }\;p^{\mu }p^{\nu }\;{\left(1+\;\frac{I}{m\;c^{2}}\right)}^{2}\;,$$

where $\lambda ,\lambda_{\mu },\lambda_{\mu \nu }$ are the Lagrange multipliers.

Hereafter, recalling the following decomposition of the particle number vector and the energy-momentum tensor

(22) $$\begin{array}{c} V^{\alpha }=\rho U^{\alpha }\;,\;T^{\alpha \beta }=\frac{e}{c^{2}}\;U^{\alpha }U^{\beta }+\;\left(p\;+\;\Pi \right)h^{\alpha \beta }+\frac{1}{c^{2}}\left(U^{\alpha }q^{\beta }+U^{\beta }q^{\alpha }\right)+t^{<\alpha \beta {>}_{3}}\;, \end{array}$$

we can choose as fields, as usual, 14 physical variables; ρ, *T*, $U^{\alpha }$, Π, $q^{\alpha }$, $t^{<\alpha \beta {>}_{3}}$, where Π is the dynamic pressure, $q^{\alpha }=-h_{\mu }^{\alpha }U_{\nu }T^{\mu \nu }$ is the heat flux, and $t^{<\alpha \beta {>}_{3}}=T^{\mu \nu }\left(h_{\mu }^{\alpha }h_{\nu }^{\beta }-\frac{1}{3}h^{\alpha \beta }h_{\mu \nu }\right)$ is the deviatoric shear viscous stress tensor. We also recall the constraints:

$$U^{\alpha }U_{\alpha }=c^{2},\;q^{\alpha }U_{\alpha }=0,\;t^{<\alpha \beta {>}_{3}}U_{\alpha }=0,\;t_{\;\;\;\;\;\;\alpha {>}_{3}}^{<\alpha }=0,$$

and we choose as the 15th variable:

(23) $$\begin{array}{c} \Delta =\frac{4}{c^{2}}\;U_{\alpha }U_{\beta }U_{\gamma }\;\left(A^{\alpha \beta \gamma }\;-\;A_{E}^{\alpha \beta \gamma }\right). \end{array}$$

The pressure *p* and the energy *e* as function of (ρ,T) are given in (12).

**Remark** **1.** *For any symmetric tensor $M^{\alpha \beta }$, we can define its traceless part $M^{<\alpha \beta >}$ and its 3-dimensional traceless part $M^{<\alpha \beta {>}_{3}}$, which is the traceless part of its projection in the 3-dimensional space orthogonal to $U^{\alpha }$, as follows*

$$\begin{array}{c} \begin{array}{cc} \; & M^{<\alpha \beta >}=\left(g_{\mu }^{\alpha }\;g_{\nu }^{\beta }-\;\frac{1}{4}\;g^{\alpha \beta }g_{\mu \nu }\right)\;M^{\mu \nu }=M^{\alpha \beta }-\;\frac{1}{4}\;g_{\mu \nu }\;M^{\mu \nu }g^{\alpha \beta }\;,\\ \; & M^{<\alpha \beta {>}_{3}}=\left(h_{\mu }^{\alpha }\;h_{\nu }^{\beta }-\;\frac{1}{3}\;h^{\alpha \beta }h_{\mu \nu }\right)\;M^{\mu \nu }\;, \end{array} \end{array}$$

*which are different except for the case in which $M^{\mu \nu }U_{\mu }=0$ and $M^{\mu \nu }g_{\mu \nu }=0$. In fact, these conditions indicate that*

$$\begin{array}{c} M^{<\alpha \beta >}=M^{<\alpha \beta {>}_{3}}\;. \end{array}$$

*Moreover, in the following, a parenthesis between two indexes indicates the symmetric part.*

### 3.1. The Linear Deviation from Equilibrium

The thermodynamical definition of the equilibrium according to Müller and Ruggeri [1] is the state in which the entropy production vanishes and hence attains its minimum value. Using this definition, the theorem was proved [19,20] that the components of the Lagrange multipliers of the balance laws of nonequilibrium variables vanish, and only the five Lagrange multipliers corresponding to the equilibrium conservation laws (Euler system) remain. In the present case, we have:

(24) $$\begin{array}{c} \begin{array}{cc} \; & \lambda_{E}=-\frac{1}{T}\left(g+c^{2}\right),\;\lambda_{\mu_{E}}=\frac{U_{\mu }}{T},\;\lambda_{\mu \nu_{E}}=0, \end{array} \end{array}$$

where g=ε+p/ρ−TS is the equilibrium chemical potential. We remark that $\lambda_{E},\lambda_{\mu_{E}}$ are the components of the *main field* that symmetrize the relativistic Euler system, as was first proved by Ruggeri and Strumia (see [21]).

In the molecular RET approach, we consider, as usual, the processes near equilibrium. For this reason, we expand (21) around an equilibrium state as follows:

(25) $$\begin{array}{c} \begin{array}{cc} \; & f_{15}≃f_{E}\left(1-\frac{1}{k_{B}}\tilde{\chi }\right),\\ \; & \tilde{\chi }=m\;\left(\lambda -\lambda_{E}\right)\;+\;\left(\lambda_{\mu }-\lambda_{\mu_{E}}\right)\;p^{\mu }\;\left(1+\;\frac{I}{m\;c^{2}}\right)\;+\;\frac{1}{m}\;\lambda_{\mu \nu }\;p^{\mu }p^{\nu }\;{\left(1+\;\frac{I}{m\;c^{2}}\right)}^{2}\;. \end{array} \end{array}$$

Inserting the distribution function (25) into the moments (19), we obtain the following system:

(26) $$\begin{array}{c} \begin{array}{cc} \; & 0=V^{\alpha }-V_{E}^{\alpha }=-\frac{m}{k_{B}}\left[V_{E}^{\alpha }\left(\lambda -\lambda_{E}\right)+T_{E}^{\alpha \mu }\left(\lambda_{\mu }-\lambda_{\mu_{E}}\right)+A_{E}^{\alpha \mu \nu }\lambda_{\mu \nu }\right]\;,\\ \; & t^{<\alpha \beta {>}_{3}}+\Pi h^{\alpha \beta }+\frac{2}{c^{2}}\;U^{(\alpha }q^{\beta )}=-\frac{m}{k_{B}}\left[T_{E}^{\alpha \beta }\left(\lambda -\lambda_{E}\right)+A_{E}^{\alpha \beta \mu }\left(\lambda_{\mu }-\lambda_{\mu_{E}}\right)+A_{E}^{\alpha \beta \mu \nu }\lambda_{\mu \nu }\right]\;,\\ \; & A^{\alpha \beta \gamma }-A_{E}^{\alpha \beta \gamma }=-\frac{m}{k_{B}}\left[A_{E}^{\alpha \beta \gamma }\left(\lambda -\lambda_{E}\right)+A_{E}^{\alpha \beta \gamma \mu }\left(\lambda_{\mu }-\lambda_{\mu_{E}}\right)+A_{E}^{\alpha \beta \gamma \mu \nu }\lambda_{\mu \nu }\right]\;, \end{array} \end{array}$$

where the equilibrium values of the tensors $A_{E}^{\alpha \beta \mu },A_{E}^{\alpha \beta \mu \nu }$, and $A_{E}^{\alpha \beta \mu \nu \gamma }$ can be obtained by (14), taking j=2,3,4:

(27) $$\begin{array}{c} \begin{array}{cc} \; & A_{E}^{\alpha \beta \gamma }=\rho \;\theta_{0,2}\;U^{\alpha }U^{\beta }U^{\gamma }\;+\;\rho c^{2}\;\theta_{1,2}\;h^{(\alpha \beta }U^{\gamma )},\\ \; & A_{E}^{\alpha \beta \mu \nu }=\rho \;\theta_{0,3}\;U^{\alpha }U^{\beta }U^{\mu }U^{\nu }\;+\;\rho \;c^{2}\;\theta_{1,3}\;h^{(\alpha \beta }U^{\mu }U^{\nu )}\;+\;\rho \;c^{4}\;\theta_{2,3}\;h^{(\alpha \beta }h^{\mu \nu )},\\ \; & A_{E}^{\alpha \beta \gamma \mu \nu }=\rho \;\theta_{0,4}\;U^{\alpha }U^{\beta }U^{\gamma }U^{\mu }U^{\nu }\;+\;\rho \;c^{2}\theta_{1,4}\;h^{(\alpha \beta }U^{\gamma }U^{\mu }U^{\nu )}\;+\;\rho \;c^{4}\theta_{2,4}\;h^{(\alpha \beta }h^{\gamma \mu }U^{\nu )}\;, \end{array} \end{array}$$

with the θ’s given in (17).

The system (26) permits one to deduce the 15 Lagrange multipliers in terms of the 15 field variables, including Δ given in (23), and then we can obtain the remaining part of the tensor $A^{\alpha \beta \gamma }$.

To solve this system, we consider first Equation (26)${}_{1}$ contracted with $U_{\alpha }$, Equation (26)${}_{2}$ contracted with $U_{\alpha }\;U_{\beta }$, Equation (26)${}_{3}$ contracted with $U_{\alpha }U_{\beta }U_{\gamma }/c^{3}$, Equation (26)${}_{2}$ contracted with $h_{\alpha \beta }/3$, and (26)${}_{3}$ contracted with $U_{\alpha }h_{\beta \gamma }/\left(3\;c^{2}\right)$, obtaining the system

(28) $$\begin{array}{c} \begin{array}{cc} \; & \theta_{0,0}\;\left(\lambda -\lambda_{E}\right)+\theta_{0,1}\;U^{\mu }\left(\lambda_{\mu }-\frac{U_{\mu }}{T}\right)+\theta_{0,2}\;U^{\mu }U^{\nu }\lambda_{\mu \nu }\;+\frac{c^{2}}{3}\theta_{1,2}\;h^{\mu \nu }\lambda_{\mu \nu }=0\;,\\ \; & \theta_{0,1}\;\left(\lambda -\lambda_{E}\right)+\theta_{0,2}\;U^{\mu }\left(\lambda_{\mu }-\frac{U_{\mu }}{T}\right)+\;\theta_{0,3}\;U^{\mu }U^{\nu }\lambda_{\mu \nu }\;+\frac{c^{2}}{6}\;\theta_{1,3}\;h^{\mu \nu }\lambda_{\mu \nu }=0\;,\\ \; & \theta_{0,2}\;\left(\lambda -\lambda_{E}\right)+\;\theta_{0,3}\;U^{\mu }\left(\lambda_{\mu }-\frac{U_{\mu }}{T}\right)+\;\theta_{0,4}\;U^{\mu }U^{\nu }\lambda_{\mu \nu }\;+\frac{c^{2}}{10}\;\theta_{1,4}\;h^{\mu \nu }\lambda_{\mu \nu }=-\;\frac{k_{B}}{4\;m^{2}\;n\;c^{4}}\;\Delta \;,\\ \; & \theta_{1,1}\;\left(\lambda -\lambda_{E}\right)+\frac{1}{3}\theta_{1,2}\;U^{\mu }\left(\lambda_{\mu }-\frac{U_{\mu }}{T}\right)+\;\frac{1}{6}\theta_{1,3}\;U^{\mu }U^{\nu }\lambda_{\mu \nu }+\;\frac{5}{9}c^{2}\theta_{2,3}\;h^{\mu \nu }\lambda_{\mu \nu }=-\;\frac{k_{B}}{m^{2}nc^{2}}\;\Pi \;,\\ \; & \frac{1}{3}\theta_{1,2}\;\left(\lambda -\lambda_{E}\right)+\;\frac{1}{6}\;\theta_{1,3}\;U^{\mu }\left(\lambda_{\mu }-\frac{U_{\mu }}{T}\right)+\;\frac{1}{10}\;\theta_{1,4}\;U^{\mu }U^{\nu }\lambda_{\mu \nu }\;+\frac{c^{2}}{9}\;\theta_{2,4}\;h^{\mu \nu }\lambda_{\mu \nu }=\\ \; & \;\;\;\;\;\;\;\;\;\;\;\;\;\;\;\;\;\;\;\;=-\;\frac{k_{B}}{3m^{2}c^{4}n}\;\left(A^{\alpha \beta \gamma }-A_{E}^{\alpha \beta \gamma }\right)U_{\alpha }h_{\beta \gamma }\;. \end{array} \end{array}$$

This is a system of 5 equations in the 4 unknowns $\lambda -\lambda_{E}$, $U^{\mu }\left(\lambda_{\mu }-\frac{U_{\mu }}{T}\right)$, $U^{\mu }U^{\nu }\lambda_{\mu \nu }$, $h^{\mu \nu }\lambda_{\mu \nu }$; in order to have solutions, the determinant of the complete matrix must be zero, that is,

(29) $$\begin{array}{c} 0=\left|\begin{array}{ccccc} \theta_{0,0} & \theta_{0,1} & \theta_{0,2} & \frac{1}{3}\theta_{1,2} & 0\\ \; & \; & & \; & \\ \theta_{0,1} & \theta_{0,2} & \theta_{0,3} & \frac{1}{6}\;\theta_{1,3} & 0\\ \; & \; & & \; & \\ \theta_{0,2} & \theta_{0,3} & \theta_{0,4} & \frac{1}{10}\;\theta_{1,4} & -\;\frac{k_{B}}{4mc^{4}}\;\Delta \\ \; & \; & & \; & \\ \theta_{1,1} & \frac{1}{3}\;\theta_{1,2} & \frac{1}{6}\;\theta_{1,3} & \frac{5}{9}\;\theta_{2,3} & -\;\frac{k_{B}}{mc^{2}}\;\Pi \\ \; & \; & & \; & \\ \frac{1}{3}\;\theta_{1,2} & \frac{1}{6}\;\theta_{1,3} & \frac{1}{10}\;\theta_{1,4} & \frac{1}{9}\;\theta_{2,4} & -\;\frac{k_{B}}{3m\;c^{4}}\;\left(A^{\alpha \beta \gamma }-A_{E}^{\alpha \beta \gamma }\right)U_{\alpha }h_{\beta \gamma } \end{array}\right|\;. \end{array}$$

By defining

$$\begin{array}{cc} \; & D_{4}=\left|\begin{array}{ccccc} \theta_{0,0} & \theta_{0,1} & \theta_{0,2} & \frac{1}{3}\theta_{1,2}\\ \; & \; & & \; & \\ \theta_{0,1} & \theta_{0,2} & \theta_{0,3} & \frac{1}{6}\;\theta_{1,3}\\ \; & \; & & \; & \\ \theta_{0,2} & \theta_{0,3} & \theta_{0,4} & \frac{1}{10}\;\theta_{1,4}\\ \; & \; & & \; & \\ \theta_{1,1} & \frac{1}{3}\;\theta_{1,2} & \frac{1}{6}\;\theta_{1,3} & \frac{5}{9}\;\theta_{2,3} \end{array}\right|\;,\\ \; & N^{\Pi }=-\;\left|\begin{array}{ccccc} \theta_{0,0} & \theta_{0,1} & \theta_{0,2} & \frac{1}{3}\theta_{1,2}\\ \; & \; & & \; & \\ \theta_{0,1} & \theta_{0,2} & \theta_{0,3} & \frac{1}{6}\;\theta_{1,3}\\ \; & \; & & \; & \\ \theta_{0,2} & \theta_{0,3} & \theta_{0,4} & \frac{1}{10}\;\theta_{1,4}\\ \; & \; & & \; & \\ \frac{1}{3}\;\theta_{1,2} & \frac{1}{6}\;\theta_{1,3} & \frac{1}{10}\;\theta_{1,4} & \frac{1}{9}\;\theta_{2,4} \end{array}\right|\;,\;N^{\Delta }=\left|\begin{array}{ccccc} \theta_{0,0} & \theta_{0,1} & \theta_{0,2} & \frac{1}{3}\theta_{1,2}\\ \; & \; & & \; & \\ \theta_{0,1} & \theta_{0,2} & \theta_{0,3} & \frac{1}{6}\;\theta_{1,3}\\ \; & \; & & \; & \\ \theta_{1,1} & \frac{1}{3}\;\theta_{1,2} & \frac{1}{6}\;\theta_{1,3} & \frac{5}{9}\;\theta_{2,3}\\ \; & \; & & \; & \\ \frac{1}{3}\;\theta_{1,2} & \frac{1}{6}\;\theta_{1,3} & \frac{1}{10}\;\theta_{1,4} & \frac{1}{9}\;\theta_{2,4} \end{array}\right|\;, \end{array}$$

Equation (29) gives:

(30) $$\begin{array}{c} \frac{1}{3\;c^{2}}\;\left(A^{\alpha \beta \gamma }-A_{E}^{\alpha \beta \gamma }\right)U_{\alpha }h_{\beta \gamma }=-\;\frac{N^{\Pi }}{D_{4}}\;\Pi \;-\;\frac{N^{\Delta }}{D_{4}}\frac{1}{4c^{2}}\;\Delta \;. \end{array}$$

We contract now Equation (26)${}_{1}$ with $h_{\alpha }^{\delta }$, Equation (26)${}_{2}$ with $U_{\alpha }\;h_{\beta }^{\delta }$, Equation (26)${}_{3}$ with $U_{\alpha }U_{\beta }h_{\gamma }^{\delta }/c^{3}$ and (26)${}_{3}$ with $h_{\alpha }^{\delta }h_{\beta \gamma }/\left(3\;c^{2}\right)$, obtaining the system

(31) $$\begin{array}{c} \begin{array}{cc} \; & c^{2}\theta_{1,1}\;h^{\delta \mu }\left(\lambda_{\mu }-\lambda_{\mu_{E}}\right)+\frac{2}{3}c^{2}\theta_{1,2}\;\;U^{\mu }h^{\delta \nu }\lambda_{\mu \nu }=0\;,\\ \; & c^{2}\theta_{1,2}\;h^{\delta \mu }\left(\lambda_{\mu }-\lambda_{\mu_{E}}\right)+\;c^{2}\theta_{1,3}\;\;U^{\mu }h^{\delta \nu }\lambda_{\mu \nu }=-\frac{3\;k_{B}}{m^{2}c^{2}n}\;q^{\delta }\;,\\ \; & c^{2}\theta_{1,3}\;h^{\delta \mu }\left(\lambda_{\mu }-\lambda_{\mu_{E}}\right)+\frac{18}{15}\;c^{2}\theta_{1,4}\;\;U^{\mu }h^{\delta \nu }\lambda_{\mu \nu }=\frac{6\;k_{B}}{m^{2}c^{4}n}\;\left(A^{\alpha \beta \gamma }-A_{E}^{\alpha \beta \gamma }\right)U_{\alpha }U_{\beta }h_{\gamma }^{\delta }\;,\\ \; & \frac{5}{3}\;c^{4}\theta_{2,3}\;h^{\delta \mu }\left(\lambda_{\mu }-\lambda_{\mu_{E}}\right)+\frac{2}{3}c^{4}\theta_{2,4}\;\;U^{\mu }h^{\delta \nu }\lambda_{\mu \nu }=\frac{k_{B}}{m^{2}n}\;\left(A^{\alpha \beta \gamma }-A_{E}^{\alpha \beta \gamma }\right)h_{\alpha \beta }h_{\gamma }^{\delta }\;. \end{array} \end{array}$$

By eliminating the parameters $h^{\delta \mu }\left(\lambda_{\mu }-\lambda_{\mu_{E}}\right)$ and $U^{\mu }h^{\delta \nu }\lambda_{\mu \nu }$ from these equations, we obtain

(32) $$\begin{array}{c} \begin{array}{cc} \; & \left(A^{\alpha \beta \gamma }-A_{E}^{\alpha \beta \gamma }\right)U_{\alpha }U_{\beta }h_{\gamma }^{\delta }=-\;c^{2}\frac{N_{3}}{D_{3}}\;q^{\delta }\;,\\ \; & \left(A^{\alpha \beta \gamma }-A_{E}^{\alpha \beta \gamma }\right)h_{\alpha \beta }h_{\gamma }^{\delta }=-\;\frac{N_{31}}{D_{3}}\;q^{\delta }\;, \end{array} \end{array}$$

with

$$\begin{array}{c} D_{3}=\left|\begin{array}{cc} \theta_{1,1} & \theta_{1,2}\\ \; & \\ \theta_{1,2} & \frac{3}{2}\;\theta_{1,3}\; \end{array}\right|\;,\;N_{3}=\frac{1}{2}\;\left|\begin{array}{cc} \theta_{1,1} & \theta_{1,2}\\ \; & \\ \theta_{1,3} & \frac{9}{5}\;\theta_{1,4}\; \end{array}\right|\;,\;N_{31}=\left|\begin{array}{cc} \theta_{1,1} & \theta_{1,2}\\ \; & \\ 5\;\theta_{2,3} & 3\;\theta_{2,4}\; \end{array}\right|\;. \end{array}$$

We contract now Equation (26)${}_{2}$ with $h_{\alpha }^{<\delta }\;h_{\beta }^{\theta {>}_{3}}$ and (26)${}_{3}$ with $h_{\alpha }^{<\delta }\;h_{\beta }^{\theta {>}_{3}}U_{\gamma }$, obtaining

(33) $$\begin{array}{c} \begin{array}{cc} \; & -\;\frac{k_{B}}{m}\;t^{<\delta \theta {>}_{3}}=\frac{2}{3}\;mnc^{4}\theta_{2,3}\;h^{\mu <\delta }h^{\theta {>}_{3}\nu }\lambda_{\mu \nu }\;,\\ \; & \left(A^{\alpha \beta \gamma }-A_{E}^{\alpha \beta \gamma }\right)h_{\alpha }^{<\delta }\;h_{\beta }^{\theta {>}_{3}}U_{\gamma }=-\;\frac{2}{15}\;\frac{m}{k_{B}}\;mn\;c^{6}\;\theta_{2,4}\;h^{\mu <\delta }h^{\theta {>}_{3}\nu }\lambda_{\mu \nu }\;, \end{array} \end{array}$$

from which it follows

(34) $$\begin{array}{c} \left(A^{\alpha \beta \gamma }-A_{E}^{\alpha \beta \gamma }\right)h_{\alpha }^{<\delta }\;h_{\beta }^{\theta {>}_{3}}U_{\gamma }=C_{5}\;c^{2}\;t^{<\delta \theta {>}_{3}}\;\;with\;C_{5}=\frac{1}{5}\;\frac{\theta_{2,4}}{\theta_{2,3}}\;. \end{array}$$

Finally, (26)${}_{3}$ contracted with $h_{\alpha }^{<\delta }\;h_{\beta }^{\theta }\;h_{\gamma }^{\psi {>}_{3}}$ gives

$$\begin{array}{c} \left(A^{\alpha \beta \gamma }-A_{E}^{\alpha \beta \gamma }\right)h_{\alpha }^{<\delta }\;h_{\beta }^{\theta }\;h_{\gamma }^{\psi {>}_{3}}=0\;. \end{array}$$

This result, jointly with (30), (32), and (34), gives the decomposition of the triple tensor $A^{\alpha \beta \gamma }$:

$$\begin{array}{c} \begin{array}{cc} A^{\alpha \beta \gamma }-A_{E}^{\alpha \beta \gamma } & =\frac{1}{4c^{4}}\;\Delta \;U^{\alpha }U^{\beta }U^{\gamma }-\;\frac{3}{4c^{2}}\;\frac{N^{\Delta }}{D_{4}}\;\Delta \;h^{(\alpha \beta }U^{\gamma )}-3\;\frac{N^{\Pi }}{D_{4}}\;\Pi h^{(\alpha \beta }U^{\gamma )}\\ \; & +\frac{3}{c^{2}}\frac{N_{3}}{D_{3}}\;q^{(\alpha }U^{\beta }U^{\gamma )}+\frac{3}{5}\frac{N_{31}}{D_{3}}h^{(\alpha \beta }q^{\gamma )}+3C_{5}t^{(<\alpha \beta {>}_{3}}U^{\gamma )}\;. \end{array} \end{array}$$

Thanks to Equation (27)${}_{1}$, we have the closure of the triple tensor in terms of the physical variables:

(35) $$\begin{array}{c} \begin{array}{cc} A^{\alpha \beta \gamma } & =\left(\rho \;\theta_{0,2}+\;\frac{1}{4c^{4}}\;\Delta \right)U^{\alpha }U^{\beta }U^{\gamma }+\left(\rho \;c^{2}\;\theta_{1,2}-\;\frac{3}{4c^{2}}\;\frac{N^{\Delta }}{D_{4}}\;\Delta \;-3\;\frac{N^{\Pi }}{D_{4}}\;\Pi \right)\;h^{(\alpha \beta }U^{\gamma )}\\ \; & +\frac{3}{c^{2}}\frac{N_{3}}{D_{3}}\;q^{(\alpha }U^{\beta }U^{\gamma )}+\frac{3}{5}\frac{N_{31}}{D_{3}}h^{(\alpha \beta }q^{\gamma )}+3C_{5}t^{(<\alpha \beta {>}_{3}}U^{\gamma )}\;. \end{array} \end{array}$$

### 3.2. Inversion of the Lagrange Multipliers

In this section, we present the explicit expression of the Lagrange multipliers in terms of the 15 physical independent variables. From the representation theorems, they are expressed as follows:

(36) $$\begin{array}{c} \begin{array}{cc} \lambda -\lambda_{E} & =a_{1}\Pi +a_{2}\Delta ,\\ \lambda_{\mu }-\lambda_{\mu_{E}} & =\left(b_{1}\Pi +b_{2}\Delta \right)U_{\mu }+b_{3}q_{\mu },\\ \lambda_{\mu \nu } & =\left(\alpha_{1}\Pi +\beta_{1}\Delta \right)U_{\mu }U_{\nu }+\left(\alpha_{2}\Pi +\beta_{2}\Delta \right)h_{\mu \nu }+\alpha_{3}\left(q_{\mu }U_{\nu }+q_{\nu }U_{\mu }\right)+\alpha_{4}t_{<\mu \nu {>}_{3}}, \end{array} \end{array}$$

where $\lambda_{E}$ and $\lambda_{\mu_{E}}$ can be found in Equation (24), and the coefficients $a_{1,2},b_{1,2,3}$, $\alpha_{1,2,3,4}$ and $\beta_{1,2}$ are functions of ρ and γ. By using Equations (28), (31) and (33), it is possible to obtain the explicit expressions of these coefficients.

For convenience, let us denote by $D_{4}^{ij}$ the minor determinant obtained from $D_{4}$ by deleting its *i*th row and *j*th column. From system (28), we obtain

(37) $$\begin{array}{c} \begin{array}{cc} \lambda -\lambda_{E} & =-\frac{k_{B}}{mc^{4}\rho \;D_{4}}\left(-\Pi \;c^{2}D_{4}^{41}+\frac{\Delta }{4}D_{4}^{31}\right),\\ \; & \\ U^{\mu }\left(\lambda_{\mu }-\lambda_{\mu_{E}}\right) & =-\frac{k_{B}}{mc^{4}\rho \;D_{4}}\left(\Pi \;c^{2}D_{4}^{42}-\frac{\Delta }{4}D_{4}^{32}\right),\\ \; & \\ U^{\beta }U^{\gamma }\lambda_{\beta \gamma } & =-\frac{k_{B}}{mc^{4}\rho \;D_{4}}\left(-\Pi \;c^{2}D_{4}^{43}+\frac{\Delta }{4}D_{4}^{33}\right),\\ \; & \\ h^{\beta \gamma }\lambda_{\beta \gamma } & =-\frac{k_{B}}{mc^{4}\rho \;D_{4}}\left(\Pi D_{4}^{44}-\frac{\Delta }{4c^{2}}D_{4}^{34}\right). \end{array} \end{array}$$

From system (31) we obtain

(38) $$\begin{array}{c} h^{\delta \mu }\left(\lambda_{\mu }-\lambda_{\mu_{E}}\right)=\frac{3\;k_{B}\theta_{1,2}}{mc^{4}\rho \;D_{3}}q^{\delta }\;\;\mathrm{and}\;\;U^{\beta }h^{\gamma \delta }\lambda_{\beta \gamma }=-\frac{9k_{B}\theta_{1,1}}{2mc^{4}\rho \;D_{3}}q^{\delta }. \end{array}$$

Finally, from Equation (33) we have

$$\begin{array}{c} h^{\beta <\delta }h^{\theta {>}_{3}\gamma }\lambda_{\beta \gamma }=-\frac{3k_{B}}{2mc^{4}\rho \theta_{2,3}}t^{<\delta \theta {>}_{3}}, \end{array}$$

that, multiplied by $t_{<\delta \theta {>}_{3}}$, gives

(39) $$\begin{array}{c} t^{<\beta \gamma {>}_{3}}\lambda_{\beta \gamma }=-\frac{3k_{B}}{2mc^{4}\rho \theta_{2,3}}t^{<\beta \gamma {>}_{3}}t_{<\beta \gamma {>}_{3}}. \end{array}$$

By comparing Equations (36)${}_{1}$ with (37)${}_{1}$, we have

(40) $$\begin{array}{c} a_{1}=\frac{k_{B}}{mc^{2}\rho \;D_{4}}D_{4}^{41},\;\;a_{2}=-\frac{k_{B}}{4\;mc^{4}\rho \;D_{4}}D_{4}^{31}. \end{array}$$

By multiplying Equation (36)${}_{2}$ times $U^{\mu }$ and $h^{\mu \delta }$, respectively, and using Equations (37)${}_{2}$ and (38)${}_{1}$, we have

(41) $$\begin{array}{c} b_{1}=-\frac{k_{B}}{mc^{4}\rho \;D_{4}}D_{4}^{42},\;\;b_{2}=\frac{k_{B}}{4\;mc^{6}\rho \;D_{4}}D_{4}^{32},\;\;b_{3}=-\frac{3\;k_{B}\theta_{1,2}}{mc^{4}\rho D_{3}}. \end{array}$$

Finally, by multiplying Equation (36)${}_{3}$ times $U^{\mu }\;U^{\nu }$, $h^{\mu \nu }$, $U^{\nu }h^{\mu \delta }$, $h^{\mu <\delta }h^{\theta >\nu }$, respectively, and using Equations (37)–(39), we obtain that

(42) $$\begin{array}{c} \begin{array}{cc} \alpha_{1} & =\frac{k_{B}}{mc^{6}\rho \;D_{4}}D_{4}^{43},\;\;\alpha_{2}=-\frac{k_{B}}{3mc^{4}\rho \;D_{4}}D_{4}^{44},\\ \alpha_{3} & =\frac{9k_{B}\theta_{1,1}}{2mc^{6}\rho \;D_{3}},\;\;\;\alpha_{4}=-\frac{3k_{B}}{2mc^{4}\rho \theta_{2,3}},\\ \beta_{1} & =-\frac{k_{B}}{4mc^{8}\rho \;D_{4}}D_{4}^{33},\;\;\beta_{2}=\frac{k_{B}}{12mc^{6}\rho \;D_{4}}D_{4}^{34}. \end{array} \end{array}$$

### 3.3. Production Term with a Variant BGK Model

To complete the closure of the system (18), we need to have the expression of the production tensor $I^{\beta \gamma }$. It depends on the collisional term *Q* (see (19)${}_{2}$), and obtaining the expression of *Q* is a hard task in relativity. Usually, for monatomic gas, the relativistic generalization of the BGK approximation first made by Marle [22,23] and successively by Anderson and Witting [24] is adopted. The Marle model is an extension of the classical BGK model in the Eckart frame [6,25], and the Anderson–Witting model obtains such extension using the Landau–Lifshitz frame [6,26]. There are some weak points for the Marle model, and the Anderson–Witting model uses the Landau–Lifshitz four velocity. Starting from these considerations, Pennisi and Ruggeri proposed a variant of the Anderson–Witting model in the Eckart frame both for monatomic and polyatomic gases, and proved that the conservation laws of particle number and energy-momentum are satisfied and the H-theorem holds [14] (see also [2]). In the polyatomic case, the following collision term has been proposed:

(43) $$\begin{array}{c} Q=\frac{U^{\alpha }p_{\alpha }}{c^{2}\tau }\left(f_{E}-f-f_{E}p^{\mu }q_{\mu }\frac{1+\frac{I}{mc^{2}}}{bmc^{2}}\right), \end{array}$$

where 3b is the coefficient of $h^{(\alpha \beta }U^{\gamma )}$ in Equation (27)${}_{1}$, that is, $3b=\rho c^{2}\theta_{1,2}$, and τ>0 denotes the relaxation time.

Recently, the existence and asymptotic behavior of classical solutions for the Boltzmann–Chernikov Equation (1) with *Q* given by (43) when the initial data is sufficiently close to a global equilibrium was proved [27].

The most general expression of a nonequilibrium double tensor as a linear function of Δ, Π, $t^{<\mu \nu {>}_{3}}$ and $q^{\mu }$ is the following:

$$\begin{array}{c} I^{\beta \gamma }=\left(B_{1}^{\Delta }\;\Delta +\;B_{1}^{\Pi }\;\Pi \right)\;U^{\beta }U^{\gamma }\;+\left(B_{2}^{\Delta }\;\Delta \;+\;B_{2}^{\Pi }\;\Pi \right)h^{\beta \gamma }+\;B^{q}\;U^{(\beta }\;q^{\gamma )}\;+\;B^{t}\;t^{<\beta \gamma {>}_{3}}. \end{array}$$

In order to determine the coefficients in $I^{\alpha \beta }$, we have to substitute Equation (43) into Equation (19)${}_{4}$, obtaining

$$\begin{array}{cc} I^{\beta \gamma } & =\frac{c}{m}\int_{R^{3}}\int_{0}^{+\infty }\frac{U^{\alpha }p_{\alpha }}{c^{2}\tau }\left(f_{E}-f-f_{E}p^{\mu }q_{\mu }\frac{1+\frac{I}{mc^{2}}}{bmc^{2}}\right)\;p^{\beta }p^{\gamma }\;{\left(1+\;\frac{I}{m\;c^{2}}\right)}^{2}\;\phi \left(I\right)\;\;d\;IdP=\\ \; & =\frac{U_{\alpha }}{c^{2}\tau }\left(A_{E}^{\alpha \beta \gamma }-A^{\alpha \beta \gamma }\right)-3\frac{U_{\alpha }q_{\mu }}{\theta_{1,2}m^{2}nc^{6}\tau }A_{E}^{\alpha \beta \gamma \mu }, \end{array}$$

then we have

(44) $$\begin{array}{c} \begin{array}{cc} B_{1}^{\Delta } & =-\frac{1}{4c^{4}\tau },\;B_{1}^{\Pi }=0,\;B_{2}^{\Delta }=\frac{1}{4c^{2}\tau }\;\frac{N^{\Delta }}{D_{4}},\;B_{2}^{\Pi }=\;\frac{1}{\tau }\;\frac{N^{\Pi }}{D_{4}}\\ B^{q} & =\frac{1}{c^{2}\tau }\;\left(\frac{\theta_{1,3}}{\theta_{1,2}}\;-2\frac{N_{3}}{D_{3}}\right)\;,\;B^{t}=-\;\frac{1}{\tau }\;C_{5}\;. \end{array} \end{array}$$

Therefore, the final expression of the production term $I^{\beta \gamma }$ is

(45) $$\begin{array}{c} I^{\beta \gamma }=\frac{1}{\tau }\left\{-\frac{1}{4c^{4}}\Delta \;U^{\beta }U^{\gamma }+\left(\frac{1}{4c^{2}}\frac{N^{\Delta }}{D_{4}}\Delta +\frac{N^{\Pi }}{D_{4}}\Pi \right)h^{\beta \gamma }+\left(-\frac{2}{c^{2}}\frac{N_{3}}{D_{3}}+\frac{\theta_{1,3}}{\theta_{1,2}}\frac{1}{c^{2}}\right)q^{(\beta }U^{\gamma )}-C_{5}t^{<\beta \gamma {>}_{3}}\right\} \end{array}$$

We summarize the results of this section as:

**Statement** **1.** *The closed system (18) obtained via MEP is the one for which $V^{\alpha },T^{\alpha \beta },A^{\alpha \beta \gamma },I^{\beta \gamma }$ are given explicitly in terms of the 15 fields ($\rho ,\gamma ,\Pi ,\Delta ,U^{\alpha },q^{\alpha },t^{<\alpha \beta {>}_{3}}$) using the expressions (22), (35), and (45). All coefficients are completely determined in terms of a single function ω(γ) given by Equation (12)${}_{3}$ and its derivatives up to the order 3. Observe, by taking into account (13), that the coefficients θ’s given in (17) can be formally written in terms of the internal energy ε and its derivatives.*

### 3.4. Closed System of the Field Equations and Material Derivative

It is now possible to explicitly write the differential system for the field variables using the material derivative. The relativistic material derivative of a function *f* is defined as the derivative with respect to the proper time $\bar{\tau }$ along the path of the particle:

(46) $$\begin{array}{c} \dot{f}=\frac{df}{d\bar{\tau }}=\frac{df}{dt}\frac{dt}{d\bar{\tau }}=\Gamma \left(\partial_{t}f+v^{j}\partial_{j}f\right)=U^{\alpha }\partial_{\alpha }f, \end{array}$$

where Γ is the Lorentz factor, and we take into account that

$$U^{\alpha }=\frac{dx^{\alpha }}{d\bar{\tau }}\equiv \left(\Gamma c,\Gamma v^{j}\right),$$

where $v^{j}$ is the velocity. Now, we observe that for any balance laws, we can have the following identity:

$$\begin{array}{c} I^{\alpha_{1}\cdots \alpha_{n}}=\partial_{\alpha }\;A^{\alpha \alpha_{1}\cdots \alpha_{n}}=g_{\alpha }^{\beta }\;\partial_{\beta }\;A^{\alpha \alpha_{1}\cdots \alpha_{n}}=\left(-h_{\alpha }^{\beta }+\frac{U^{\beta }U_{\alpha }}{c^{2}}\right)\;\partial_{\beta }\;A^{\alpha \alpha_{1}\cdots \alpha_{n}}=\\ =\frac{U_{\alpha }}{c^{2}}\;{\dot{A}}^{\alpha \alpha_{1}\cdots \alpha_{n}}\;-\;h_{\alpha }^{\beta }\;\partial_{\beta }\;A^{\alpha \alpha_{1}\cdots \alpha_{n}}\;. \end{array}$$

In our case with n=0,1,2, these equations are written as follows:

$$\begin{array}{c} \begin{array}{cc} \; & \partial_{\alpha }\left(\rho U^{\alpha }\right)=0,\;\;h_{\delta \beta }\;\left(\frac{U_{\alpha }}{c^{2}}\;{\dot{T}}^{\alpha \beta }\;-\;h_{\alpha }^{\mu }\;\partial_{\mu }\;T^{\alpha \beta }\right)=0,\;\;U_{\beta }\;\left(\frac{U_{\alpha }}{c^{2}}\;{\dot{T}}^{\alpha \beta }\;-\;h_{\alpha }^{\mu }\;\partial_{\mu }\;T^{\alpha \beta }\right)=0,\\ \; & h_{\delta \beta }\;h_{\theta \gamma }\;\left(\frac{U_{\alpha }}{c^{2}}\;{\dot{A}}^{\alpha \beta \gamma }\;-\;h_{\alpha }^{\mu }\;\partial_{\mu }\;A^{\alpha \beta \gamma }\;-\;I^{\beta \gamma }\right)=0\;,\\ \; & h_{\delta \beta }\;U_{\gamma }\;\left(\frac{U_{\alpha }}{c^{2}}\;{\dot{A}}^{\alpha \beta \gamma }\;-\;h_{\alpha }^{\mu }\;\partial_{\mu }\;A^{\alpha \beta \gamma }\;-\;I^{\beta \gamma }\right)=0,\;U_{\beta }\;U_{\gamma }\;\left(\frac{U_{\alpha }}{c^{2}}\;{\dot{A}}^{\alpha \beta \gamma }\;-\;h_{\alpha }^{\mu }\;\partial_{\mu }\;A^{\alpha \beta \gamma }\;-\;I^{\beta \gamma }\right)=0\;. \end{array} \end{array}$$

By using the expressions (22), (35), and (45), respectively, for $V^{\alpha },T^{\alpha \beta }$, $A^{\alpha \beta \gamma }$ and $I^{\beta \gamma }$, we see that these become

(47) $$\begin{array}{cc} \; & \dot{\rho }+\rho \;\partial_{\alpha }\;U^{\alpha }=0\;,\\ \; & -\frac{e+p+\Pi }{c^{2}}\;{\dot{U}}^{\delta }\;+\;\frac{1}{c^{2}}\;h_{\beta }^{\delta }\;{\dot{q}}^{\beta }\;+\;\frac{1}{c^{2}}\;t^{<\alpha \delta {>}_{3}}\;{\dot{U}}_{\alpha }\;-\;h^{\delta \mu }\;\partial_{\mu }\left(p+\Pi \right)\;-\;\frac{1}{c^{2}}\;q^{\mu }\;\partial_{\mu }U^{\delta }\;-\frac{1}{c^{2}}\;q^{\delta }\;\partial_{\alpha }U^{\alpha }\;-\;h_{\beta }^{\delta }\;h_{\alpha }^{\mu }\;\partial_{\mu }\;t^{<\alpha \beta {>}_{3}}=0\;,\\ \; & \dot{e}\;+\;2\;\frac{U_{\alpha }}{c^{2}}\;{\dot{q}}^{\alpha }\;+\;\left(e+p+\Pi \right)\;\partial_{\alpha }U^{\alpha }\;-\;h_{\alpha }^{\mu }\;\partial_{\mu }q^{\alpha }\;-t^{<\alpha \beta {>}_{3}}\;\partial_{\alpha }U_{\beta }=0\;,\\ \; & h_{\delta \beta }\;{\left(\frac{1}{3}\rho c^{2}\theta_{1,2}\;-\;\frac{1}{4\;c^{2}}\;\frac{N^{\Delta }}{D_{4}}\;\Delta \;-\;\frac{N^{\Pi }}{D_{4}}\;\Pi \right)}^{•}+\;C_{5}\;h_{\delta \gamma }\;h_{\theta \beta }\;\dot{t}{\;}^{<\theta \gamma {>}_{3}}\;+\;t_{<\delta \beta {>}_{3}}\;{\dot{C}}_{5}-\frac{2}{c^{2}}\;\left(\frac{N_{3}}{D_{3}}\;+\;\frac{1}{5}\;\frac{N_{31}}{D_{3}}\right)q_{(\delta }\;h_{\beta )\gamma }\;{\dot{U}}^{\gamma }\;-\\ \; & \;\frac{1}{5\;c^{2}}\;\frac{N_{31}}{D_{3}}\;h_{\beta \delta }\;q^{\alpha }\;{\dot{U}}_{\alpha }+\;\left(-\;\frac{1}{3}\rho c^{2}\theta_{1,2}\;+\;\frac{1}{4\;c^{2}}\;\frac{N^{\Delta }}{D_{4}}\;\Delta \;+\;\frac{N^{\Pi }}{D_{4}}\;\Pi \right)\;\left[-h_{\delta \beta }\partial_{\alpha }\;U^{\alpha }\;+\;2\;h_{\theta (\delta }\;h_{\beta )}^{\mu }\;\partial_{\mu }\;U^{\theta }\right]\;+\\ \; & \;\frac{1}{5}\;\left(q^{\mu }h_{\delta \beta }+2\;q_{(\delta }h_{\beta )}^{\mu }\right)\;\partial_{\mu }\;\left(\frac{N_{31}}{D_{3}}\right)-\;\frac{1}{5}\;\frac{N_{31}}{D_{3}}\;\left[h_{\delta \beta }\;h_{\alpha }^{\mu }\;\partial_{\mu }\;q^{\alpha }+2\;h_{\theta (\delta }h_{\beta )}^{\mu }\;\partial_{\mu }\;q^{\theta }\right]+\\ \; & \;C_{5}\;\left[\;t_{<\delta \beta {>}_{3}}\;\partial_{\alpha }\;U^{\alpha }\;+\;2\;t^{<\mu \gamma {>}_{3}}h_{\gamma (\beta }\;h_{\delta )\theta }\;\partial_{\mu }\;U^{\theta }\right]=\\ \; & \;\frac{1}{\tau }\;\left(\frac{1}{4c^{2}}\;\frac{N^{\Delta }}{D_{4}}\;\Delta \;+\;\frac{N^{\Pi }}{D_{4}}\;\Pi \right)h_{\delta \beta }\;-\;\frac{1}{\tau }\;C_{5}\;t_{<\delta \beta {>}_{3}}\;,\\ \; & h_{\beta \delta }\;{\dot{U}}^{\beta }\left(\rho \theta_{0,2}c^{2}+\frac{2}{3}\rho c^{2}\theta_{1,2}+\frac{1}{4\;c^{2}}\;\Delta -\;\frac{1}{2\;c^{2}}\;\frac{N^{\Delta }}{D_{4}}\;\Delta -\;2\;\frac{N^{\Pi }}{D_{4}}\;\Pi \right)+h_{\beta \delta }\;\frac{N_{3}}{D_{3}}\;{\dot{q}}^{\beta }-\;q_{\delta }\;{\left(\frac{N_{3}}{D_{3}}\right)}^{•}\;+\;\left(2\;C_{5}\;-1\right)\;t_{<\delta \gamma {>}_{3}}\;{\dot{U}}^{\gamma }\;-\\ \; & \;h_{\delta }^{\mu }\;\partial_{\mu }\;\left(\frac{1}{3}\rho c^{4}\theta_{1,2}-\frac{1}{4}\;\frac{N^{\Delta }}{D_{4}}\;\Delta -\;\frac{N^{\Pi }}{D_{4}}\;c^{2}\;\Pi \right)-\left(\frac{N_{3}}{D_{3}}\;+\;\frac{1}{5}\;\frac{N_{31}}{D_{3}}\right)\;\left(q^{\mu }\;\partial_{\mu }\;U_{\delta }\;+q_{\delta }\;\partial_{\alpha }\;U^{\alpha }\right)+\\ \; & \;\frac{1}{5}\;\frac{N_{31}}{D_{3}}\;h_{\delta }^{\mu }\;q^{\gamma }\;\partial_{\mu }\;U_{\gamma }\;+\;h^{\alpha \mu }\;\partial_{\mu }\;\left(C_{5}\;c^{2}\;t_{<\alpha \delta {>}_{3}}\right)=\frac{1}{\tau }\;\left(\frac{N_{3}}{D_{3}}\;-\;\frac{\theta_{1,3}}{2\;\theta_{1,2}}\right)\;q_{\delta }\;,\\ \; & {\left(\rho \theta_{0,2}c^{4}+\frac{1}{4}\;\Delta \right)}^{•}-\;3\;\frac{N_{3}}{D_{3}}\;q^{\alpha }\;{\dot{U}}_{\alpha }\;+\partial_{\alpha }\;U^{\alpha }\cdot \left(\rho \theta_{0,2}c^{4}+\frac{2}{3}\rho c^{4}\theta_{1,2}+\frac{1}{4}\;\Delta -\;\frac{1}{2}\;\frac{N^{\Delta }}{D_{4}}\;\Delta -\;2\;\frac{N^{\Pi }}{D_{4}}\;\Pi \;c^{2}\right)\;-\\ \; & \;h_{\alpha }^{\mu }\;\partial_{\mu }\;\left(\frac{N_{3}}{D_{3}}\;c^{2}\;q^{\alpha }\right)\;-\;2\;C_{5}c^{2}\;t^{<\mu \gamma {>}_{3}}\;\partial_{\mu }U_{\gamma }=-\;\frac{1}{4\;\tau }\;\Delta \;. \end{array}$$

It may be useful to decompose (47)${}_{4}$ into the trace and spatial traceless parts. The trace part is given by

(48) $$\begin{array}{c} \begin{array}{cc} \; & \;{\left(\rho c^{2}\theta_{1,2}\;-\;\frac{3}{4\;c^{2}}\;\frac{N^{\Delta }}{D_{4}}\;\Delta \;-\;3\frac{N^{\Pi }}{D_{4}}\;\Pi \right)}^{•}+C_{5}h_{\theta \gamma }\dot{t}{\;}^{<\theta \gamma {>}_{3}}+\frac{1}{c^{2}}\;\left(2\frac{N_{3}}{D_{3}}\;-\frac{1}{5}\frac{N_{31}}{D_{3}}\right)q_{\gamma }\;{\dot{U}}^{\gamma }\;-\\ \; & \;\;\left(-\;\frac{1}{3}\rho c^{2}\theta_{1,2}\;+\;\frac{1}{4\;c^{2}}\;\frac{N^{\Delta }}{D_{4}}\;\Delta \;+\;\frac{N^{\Pi }}{D_{4}}\;\Pi \right)\;\partial_{\alpha }\;U^{\alpha }\;+q^{\mu }\partial_{\mu }\;\left(\frac{N_{31}}{D_{3}}\right)\;\;-\\ \; & \;\;\frac{N_{31}}{D_{3}}\;h_{\alpha }^{\mu }\;\partial_{\mu }\;q^{\alpha }-2C_{5}\;t_{\;\;\;\;\;\gamma {>}_{3}}^{<\mu }\partial_{\mu }\;U^{\gamma }=\frac{3}{\tau }\;\left(\frac{1}{4c^{2}}\;\frac{N^{\Delta }}{D_{4}}\;\Delta \;+\;\frac{N^{\Pi }}{D_{4}}\;\Pi \right), \end{array} \end{array}$$

and the spatial traceless part is:

(49) $$\begin{array}{c} \begin{array}{cc} \; & C_{5}\;h_{\gamma <\delta }\;h_{\beta {>}_{3}\theta }{\dot{t}}^{<\gamma \theta {>}_{3}}\;+\;t_{<\delta \beta {>}_{3}}\;{\dot{C}}_{5}\;+\;\frac{2}{c^{2}}\;\left(\frac{N_{3}}{D_{3}}\;+\;\frac{1}{5}\;\frac{N_{31}}{D_{3}}\right)q_{<\delta }\;{\dot{U}}_{\beta {>}_{3}}\;+\\ \; & +\;2\;\left(-\;\frac{1}{3}\;\rho \;c^{2}\theta_{1,2}\;+\;\frac{1}{4\;c^{2}}\;\frac{N^{\Delta }}{D_{4}}\;\Delta \;+\;\frac{N^{\pi }}{D_{4}}\;\pi \right)\;h_{\gamma <\delta }\;h_{\;\;\beta {>}_{3}}^{\mu }\;\partial_{\mu }\;U^{\gamma }\;+\\ \; & +\frac{2}{5}\;\left(q_{<\delta }h_{\;\;\beta {>}_{3}}^{\mu }\right)\;\partial_{\mu }\;\left(\frac{N_{31}}{D_{3}}\right)-\;\frac{2}{5}\;\frac{N_{31}}{D_{3}}\;\left(h_{\gamma <\delta }h_{\;\;\beta {>}_{3}}^{\mu }\;\partial_{\mu }\;q^{\gamma }\right)+\\ \; & +C_{5}\;\left[t_{<\delta \beta {>}_{3}}\;\partial_{\alpha }\;U^{\alpha }\;+\;2\;t^{<\mu \gamma {>}_{3}}h_{\gamma <\beta }\;h_{\delta {>}_{3}\nu }\;\partial_{\mu }\;U^{\nu }\right]=-\;\frac{1}{\tau }\;C_{5}\;t_{<\delta \beta {>}_{3}}\;. \end{array} \end{array}$$

The system formed by the 15 Equations (47)${}_{1,2,3}$, (48), (49) and (47)${}_{5,6}$ is a closed system for the 15 unknown $\left(\rho ,U_{\delta },T,\Pi ,t_{<\alpha \beta {>}_{3}},q_{\delta },\Delta \right)$.

## 4. Entropy Density, Convexity, Entropy Principle, and Well-Posedness of Cauchy Problem

In this section, we evaluate the entropy law, and we want to prove that all solutions are entropic with an entropy density that is a convex function.

### 4.1. Entropy Density

By substituting the distribution function (25) with (36) into (20), we can evaluate the four-dimensional entropy flux. In this procedure, it is necessary to be careful concerning the order of the nonequilibrium variables. The present linear constitutive equation is related to the entropy with the second order of the nonequilibrium variables. By taking into account up to the second order in the expansion of the distribution function and of the constitutive equations, we may evaluate as follows:

(50) $$\begin{array}{c} h^{\alpha }=h_{E}^{\alpha }\;+\;h_{\left(1\right)}^{\alpha }\;+\;h_{\left(2\right)}^{\alpha }\;, \end{array}$$

where $h_{\left(1\right)}^{\alpha }$ and $h_{\left(2\right)}^{\alpha }$ are, respectively, the contribution of the first and second order terms of the nonequilibrium variables, which can be derived as follows (see Appendix A for details):

(51) $$\begin{array}{c} \begin{array}{cc} h_{\left(1\right)}^{\alpha } & =-\frac{c}{k_{B}}\int_{R^{3}}\int_{0}^{+\infty }p^{\alpha }f_{E}\;\chi_{E}\;{\tilde{\chi }}_{\left(1\right)}\;\varphi \left(I\right)\;dI\;dP,\\ h_{\left(2\right)}^{\alpha } & =-\frac{c}{2\;k_{B}}\int_{R^{3}}\int_{0}^{+\infty }p^{\alpha }f_{E}\;{\tilde{\chi }}_{\left(1\right)}{}^{2}\;\varphi \left(I\right)\;dI\;dP\;, \end{array} \end{array}$$

where ${\tilde{\chi }}_{\left(1\right)}$ is $\tilde{\chi }$ defined in (25) with the linear constitutive equations studied in the previous. After cumbersome calculations, we obtain explicit expression of them as follows:

(52) $$\begin{array}{cc} h_{\left(1\right)}^{\alpha } & =\lambda_{E}\left(V^{\alpha }-V_{E}^{\alpha }\right)\;+\;\frac{U_{\mu }}{T}\;\left(T^{\alpha \mu }-T_{E}^{\alpha \mu }\right)=\frac{q^{\alpha }}{T},\\ h_{\left(2\right)}^{\alpha } & =-\frac{m}{2\;k_{B}}\left\{{\left[\left(\lambda -\lambda^{E}\right)\right]}^{2}V_{E}^{\alpha }\;+\;\left(\lambda_{\mu }-\lambda_{\mu }^{E}\right)\left(\lambda_{\nu }-\lambda_{\nu }^{E}\right)\;A_{E}^{\alpha \mu \nu }\;+\;\left(\lambda_{\mu \nu }\right)\left(\lambda_{\psi \theta }\right)\;A_{E}^{\alpha \mu \nu \psi \theta }\;+\right.\\ \; & \;+\left.2\left(\lambda -\lambda^{E}\right)\left(\lambda_{\mu }-\lambda_{\mu }^{E}\right)\;T_{E}^{\alpha \mu }\;+\;2\left(\lambda -\lambda^{E}\right)\left(\lambda_{\mu \nu }\right)\;A_{E}^{\alpha \mu \nu }\;+\;2\left(\lambda_{\theta }-\lambda_{\theta }^{E}\right)\left(\lambda_{\mu \nu }\right)\;A_{E}^{\alpha \theta \mu \nu }\right\}\;\\ \; & =-\frac{1}{c^{2}}U^{\alpha }\left\{-\frac{c^{2}\alpha_{4}C_{5}}{2}t^{<\mu \nu {>}_{3}}t_{<\mu \nu {>}_{3}}-\left(c^{2}\alpha_{3}\frac{N_{3}}{D_{3}}+\frac{b_{3}}{2}\right)q_{\mu }q^{\mu }+L_{1}\Pi^{2}+L_{2}\Delta^{2}+2L_{3}\Pi \Delta \right\}\\ \; & \;+\frac{1}{2}\left(b_{1}-b_{3}+c^{2}\frac{N_{3}}{D_{3}}\alpha_{1}+\frac{N_{31}}{D_{3}}\alpha_{2}+2\alpha_{3}c^{2}\frac{N^{\Pi }}{D_{4}}\right)\Pi q^{\alpha }+\frac{1}{2}\left(b_{2}+c^{2}\frac{N_{3}}{D_{3}}\beta_{1}+\frac{N_{31}}{D_{3}}\beta_{2}+\frac{1}{2}\alpha_{3}\frac{N^{\Delta }}{D_{4}}\right)\Delta q^{\alpha }\\ \; & \;+\frac{1}{2}\left(b_{3}+2c^{2}\alpha_{3}C_{5}-\frac{2}{5}\alpha_{4}\frac{N_{31}}{D_{3}}\right)t^{<\alpha \mu {>}_{3}}q_{\mu }, \end{array}$$

where

$$\begin{array}{cc} \; & L_{1}=\frac{3c^{2}}{2}\alpha_{2}\frac{N^{\Pi }}{D_{4}},\;L_{2}=\frac{1}{8}\left(\;3\beta_{2}\frac{N^{\Delta }}{D_{4}}-c^{2}\beta_{1}\right),\;L_{3}=\frac{1}{4}\left(\frac{3\alpha_{2}}{4}\frac{N^{\Delta }}{D_{4}}+3c^{2}\beta_{2}\frac{N^{\Pi }}{D_{4}}-\frac{c^{2}\alpha_{1}}{4}\right). \end{array}$$

In particular, for the entropy density $h=h^{\alpha }U_{\alpha }$, we have

(53) $$\begin{array}{c} h=h_{E}+\frac{c^{2}\alpha_{4}C_{5}}{2}t^{<\mu \nu {>}_{3}}t_{<\mu \nu {>}_{3}}+\left(c^{2}\alpha_{3}\frac{N_{3}}{D_{3}}+\frac{b_{3}}{2}\right)q_{\mu }q^{\mu }-\left(\begin{array}{cc} \Pi & \Delta \end{array}\right)\left(\begin{array}{cc} L_{1} & L_{3}\\ L_{3} & L_{2} \end{array}\right)\left(\begin{array}{c} \Pi \\ \Delta \end{array}\right). \end{array}$$

We emphasize that the convexity of the entropy density is satisfied because from (52)${}_{1}$, we have $h_{\left(1\right)}^{\alpha }U_{\alpha }=0$, and from (51), we have $h_{\left(2\right)}^{\alpha }U_{\alpha }<0$ everywhere and zero only at equilibrium. Therefore, the following inequalities are automatically satisfied:

$$\alpha_{4}C_{5}<0,\;2c^{2}\alpha_{3}\frac{N_{3}}{D_{3}}+b_{3}>0\;\;\;\left(\mathrm{because}\;\;q_{\alpha }q^{\alpha }<0\right),\;L_{1}>0,\;L_{1}\;L_{2}-{\left(L_{3}\right)}^{2}>0.$$

### 4.2. Entropy Production

According with the theorem proved by Boillat and Ruggeri [19] (see also [1,2]), the procedure of MEP at molecular level is equivalent to the closure using the entropy principle, and the Lagrange multipliers coincide with the *main field* for which the original system becomes symmetric hyperbolic [2]. Therefore, the closed system satisfies the entropy balance law

(54) $$\partial_{\alpha }h^{\alpha }=\Sigma ,$$

where the entropy four-vector is given by (50), (52). For what concerns the entropy production Σ according to the result of Ruggeri and Strumia [2], this is given by the scalar product between the main field components and the production terms [21]. In the present case, we have

(55) $$\begin{array}{c} \Sigma =I^{\beta \gamma }\;\lambda_{\beta \gamma }. \end{array}$$

By using Equation (45), we have

(56) $$\begin{array}{c} \Sigma =\frac{1}{\tau }\left\{-\frac{1}{4c^{4}}\Delta \;U^{\beta }U^{\gamma }\lambda_{\beta \gamma }+\left(\frac{1}{4c^{2}}\frac{N^{\Delta }}{D_{4}}\Delta +\frac{N^{\Pi }}{D_{4}}\Pi \right)h^{\beta \gamma }\lambda_{\beta \gamma }+\left(-\frac{2}{c^{2}}\frac{N_{3}}{D_{3}}+\frac{\theta_{1,3}}{\theta_{1,2}}\frac{1}{c^{2}}\right)q^{(\beta }U^{\gamma )}\lambda_{\beta \gamma }-C_{5}t^{<\beta \gamma {>}_{3}}\lambda_{\beta \gamma }\right\}. \end{array}$$

By substituting Equations (37)–(39) into Equation (56), and remembering that $q^{\beta }U^{\gamma }\lambda_{\beta \gamma }=-q_{\alpha }h^{\alpha \beta }U^{\gamma }\lambda_{\beta \gamma }$, we obtain Σ in a quadratic form, as follows:

(57) $$\begin{array}{c} \Sigma =\frac{3k_{B}\;C_{5}}{2\tau mc^{4}\rho \theta_{2,3}}t^{<\beta \gamma {>}_{3}}t_{<\beta \gamma {>}_{3}}+\frac{9k_{B}\theta_{1,1}}{2\tau m^{2}\;n\;c^{6}D_{3}}\left(-2\frac{N_{3}}{D_{3}}+\frac{\theta_{1,3}}{\theta_{1,2}}\right)q^{\alpha }q_{\alpha }+\left(\begin{array}{cc} \Delta & \Pi \end{array}\right)\left(\begin{array}{cc} M_{1} & M_{2}\\ M_{2} & M_{3} \end{array}\right)\left(\begin{array}{c} \Delta \\ \Pi \end{array}\right), \end{array}$$

where

$$\begin{array}{c} \begin{array}{cc} \; & M_{1}=\frac{k_{B}}{16\;c^{8}\tau m^{2}\;n\;D_{4}}\left(D_{4}^{33}+\frac{N^{\Delta }}{D_{4}}D_{4}^{34}\right),\;M_{2}=-\frac{k_{B}}{4\;c^{6}\tau m^{2}\;n\;D_{4}}\left(D_{4}^{43}+\frac{N^{\Delta }}{D_{4}}D_{4}^{44}-\frac{N^{\Pi }}{D_{4}}D_{4}^{34}\right),\\ \; & M_{3}=-\frac{k_{B}}{c^{4}\tau m^{2}\;n\;D_{4}}\frac{N^{\Pi }}{D_{4}}D_{4}^{44}. \end{array} \end{array}$$

The Sylvester criteria allow us to state that the quadratic form is positive definite iff all the following conditions hold:

(58) $$\frac{3k_{B}\;C_{5}}{2\tau mc^{4}\rho \theta_{2,3}}>0,\;\left(-2\frac{N_{3}}{D_{3}}+\frac{\theta_{1,3}}{\theta_{1,2}}\right)\frac{9k_{B}\theta_{1,1}}{2\tau m^{2}\;n\;c^{6}D_{3}}<0,\;M_{1}>0,\;M_{1}\;M_{3}-{\left(M_{2}\right)}^{2}>0.$$

The first condition of (58) is automatically satisfied because of the definition of the functions involved.

In order to prove the second condition, we can consider a space like vector $X^{\beta }$ and the following function that is defined to be positive for each value of $X^{\beta }$:

$$\begin{array}{c} g\left(X^{\beta }\right)=\frac{U_{\alpha }}{c\;\tau \;k_{B}}\int_{R^{3}}\int_{0}^{+\infty }f_{E}p^{\alpha }{\left[X_{\beta }p^{\beta }\left(\frac{\theta_{1,3}}{\theta_{1,2}}\left(1+\frac{I}{mc^{2}}\right)-\frac{2}{mc^{2}}{\left(1+\frac{I}{mc^{2}}\right)}^{2}U_{\nu }p^{\nu }\right)\right]}^{2}\phi \left(I\right)dIdP\;. \end{array}$$

By exploiting the calculation in the above integral and by using Equation (27), we have

$$\begin{array}{c} g\left(X^{\beta }\right)=\frac{m^{2}\;n\;c^{2}}{\tau \;k_{B}}\left[\frac{1}{3}\frac{{\left(\theta_{1,3}\right)}^{2}}{\theta_{1,2}}-\frac{2}{5}\theta_{1,4}\right]X^{\beta }\;X_{\beta }\;. \end{array}$$

If we choose, as a particular value,

$$\begin{array}{c} X^{\beta }=-\frac{1}{D_{3}}\frac{9\;k_{B}}{2\;m^{2}\;n\;c^{4}}\theta_{1,1}\;q^{\beta }, \end{array}$$

we obtain

$$\begin{array}{c} g\left(X^{\beta }\right)=\frac{9k_{B}\theta_{1,1}}{2\tau m^{2}\;n\;c^{6}D_{3}}\left(-2\frac{N_{3}}{D_{3}}+\frac{\theta_{1,3}}{\theta_{1,2}}\right)q^{\alpha }q_{\alpha }>0. \end{array}$$

This proves that also the second condition of (58) is satisfied.

Conditions 3 and 4 of (58) can be proved by showing that they are coefficients of a quadratic form that is definite positive. In order to obtain the entropy production up to the second order, we have to substitute Equation (19)${}_{4}$ into (55) and take the collisional term (43) up to the first order. Then,

$$\begin{array}{c} \Sigma^{\left(2\right)}=\frac{c}{m}\int_{R^{3}}\int_{0}^{+\infty }Q^{\left(1\right)}\;p^{\beta }p^{\gamma }\;\lambda_{\beta \gamma }\;{\left(1+\frac{I}{mc^{2}}\right)}^{2}\;\phi \left(I\right)\;dI\;dP, \end{array}$$

with

$$\begin{array}{c} Q^{\left(1\right)}=\frac{f_{E}}{c^{2}\;\tau \;k_{B}}U_{\alpha }p^{\alpha }\left[\tilde{\chi }-\frac{k_{B}}{bmc^{2}}p^{\mu }q_{\mu }\left(1+\frac{I}{mc^{2}}\right)\right]. \end{array}$$

If we substitute to $\lambda_{\beta \gamma }$ its expression obtained from Equation (25)${}_{2}$, we obtain

$$\begin{array}{c} \Sigma^{\left(2\right)}=c\int_{R^{3}}\int_{0}^{+\infty }Q^{\left(1\right)}\;\tilde{\chi }\;\phi \left(I\right)\;dI\;dP. \end{array}$$

In the state where $q^{\beta }=0$ and $t^{<\alpha \beta {>}_{3}}=0$, the Lagrange multipliers and the Entropy production assume particular values that we denote with a *, in particular

$$\begin{array}{c} \Sigma^{\left(2*\right)}=\frac{c}{m}\int_{R^{3}}\int_{0}^{+\infty }Q^{\left(1*\right)}\;{\tilde{\chi }}^{*}\;\phi \left(I\right)\;dI\;dP=\frac{c}{m}\int_{R^{3}}\int_{0}^{+\infty }\frac{f_{E}}{c^{2}\;\tau \;k_{B}}U_{\alpha }p^{\alpha }\;{\left[\tilde{\chi *}\right]}^{2}\phi \left(I\right)\;dI\;dP, \end{array}$$

which is clearly a positive quantity. Moreover, we have

$$\begin{array}{c} \Sigma^{\left(2*\right)}=I^{\beta \gamma *}\lambda_{\beta \gamma *} \end{array}$$

which corresponds to the quadratic form

$$\begin{array}{c} \left(\begin{array}{cc} \Delta & \Pi \end{array}\right)\left(\begin{array}{cc} M_{1} & M_{2}\\ M_{2} & M_{3} \end{array}\right)\left(\begin{array}{c} \Delta \\ \Pi \end{array}\right), \end{array}$$

which, therefore, turns out to be definite positive. Therefore, the following is proved:

**Statement** **2.** *The entropy density (53) is a convex function and has its maximum at equilibrium. The solutions satisfies the entropy principle (54) with an entropy production (57) that is always non-negative. According to the general theory of symmetrization given first in covariant formulation in [21], and the equivalence between Lagrange multipliers and main field [19], the closed system is symmetric hyperbolic in the neighborhood of equilibrium if we chose as variables the main field variables (36), with coefficients given in (40)–(42), and the Cauchy problem is well posed locally in time.*

## 5. Diatomic Gases

The system (47) is very complex, in particular, because it is not simple to evaluate the function ω(γ), which involves two integrals (12)${}_{3}$ that cannot have analytical expression for a generic polyatomic gas. Taking into account the relations [7]

$$J_{2,1}\left(\gamma \right)=\frac{1}{\gamma }K_{2}\left(\gamma \right),\;\;J_{2,2}\left(\gamma \right)=\frac{1}{\gamma }\left(K_{3}\left(\gamma \right)-\frac{1}{\gamma }K_{2}\left(\gamma \right)\right),$$

where $K_{n}$ denotes the modified Bessel function, we can rewrite ω given in (12)${}_{3}$ in terms of the modified Bessel functions [7]:

$$\omega \left(\gamma \right)=\frac{1}{\gamma }\left(\frac{\int_{0}^{+\infty }K_{3}\left(\gamma^{*}\right)\;\phi \left(I\right)\;d\;I}{\int_{0}^{+\infty }\frac{K_{2}\left(\gamma^{*}\right)}{\gamma^{*}}\phi \left(I\right)\;d\;I}-1\right).$$

Moreover, to calculate the integrals, we need to prescribe the measure ϕ(I). In [7], the measure ϕ(I) was assumed as

$$\phi \left(I\right)=I^{a},\;a=\frac{D-5}{2},$$

because it is the one for which the macroscopic internal energy in the classical limit, when γ→∞, it converges with that of a classical polyatomic gas, where *D* indicates the degree of freedom of a molecule. As was observed by Ruggeri, Xiao, and Zhao [28] in the case of a=0 (i.e., D=5 corresponding to diatomic gas), the energy *e* has an explicit expression similar to monatomic gas:

$$e=p\left(\frac{\gamma K_{0}\left(\gamma \right)}{K_{1}\left(\gamma \right)}+3\right).$$

Therefore, from (12), we have

$$\omega_{\mathrm{diat}}\left(\gamma \right)=\frac{K_{0}\left(\gamma \right)}{K_{1}\left(\gamma \right)}+\frac{3}{\gamma }.$$

Using the following recurrence formulas of the Bessel functions

(59) $$\begin{array}{c} K_{n}\left(\gamma \right)=\frac{\gamma }{2n}\left(K_{n+1}\left(\gamma \right)-K_{n-1}\left(\gamma \right)\right)\;, \end{array}$$

we can express ω in terms of

$$G\left(\gamma \right)=\frac{K_{3}\left(\gamma \right)}{K_{2}\left(\gamma \right)}.$$

In fact, we can obtain immediately the following expression:

(60) $$\begin{array}{c} \omega_{\mathrm{diat}}\left(\gamma \right)=\frac{1}{\gamma }+\frac{\gamma }{\gamma G-4}, \end{array}$$

which is a simple function similar to the one of monatomic gas, for which we have [3]:

$$\begin{array}{c} \omega_{\mathrm{mono}}\left(\gamma \right)=-1+\;\gamma \;G. \end{array}$$

Taking into account that the derivatives of the Bessel function are known, all coefficients appearing in the differential system (47) can be written explicitly in terms of G(γ), by using (59) and the recurrence Formula (58). This is simple by using a symbolic calculus like Mathematica^®^.

## 6. Ultra-Relativistic Limit

In the ultra-relativistic limit where γ→0, it was proved in [29,30] that the energy converges to

(61) $$\begin{array}{c} e=\left(\alpha +1\right)\frac{n\;m\;c^{2}}{\gamma }\;,\;with\;\alpha =\left\{\begin{array}{cc} 2 & if\;a\leq 2\\ a & \;\;\;if\;a\geq 2\;. \end{array}\right. \end{array}$$

This implies

(62) $$\begin{array}{c} \omega_{\mathrm{ultra}}=\frac{\left(\alpha +1\right)}{\gamma }\;,\;with\;\alpha =\left\{\begin{array}{cc} 2 & if\;a\leq 2\\ a & \;\;\;if\;a\geq 2\;. \end{array}\right. \end{array}$$

By means of this expression, we can evaluate the coefficients $\theta_{h,j}$ in (17), which become:

$$\begin{array}{c} \begin{array}{cc} \; & \left\{\theta_{0,0},\theta_{0,1},\theta_{0,2},\theta_{0,3},\theta_{0,4}\right\}=\\ \; & \;\left\{1,\frac{\alpha +1}{\gamma },\frac{\left(\alpha +1)(\alpha +2\right)}{\gamma^{2}},\frac{\left(\alpha +1)(\alpha +2)(\alpha +3\right)}{\gamma^{3}},\frac{\left(\alpha +1)(\alpha +2)(\alpha +3)(\alpha +4\right)}{\gamma^{4}}\right\},\\ \; & \left\{\theta_{1,1},\theta_{1,2},\theta_{1,3},\theta_{1,4}\right\}=\\ \; & \;\left\{\frac{1}{\gamma },\frac{3(\alpha +2)}{\gamma^{2}},\frac{6(\alpha +2)(\alpha +3)}{\gamma^{3}},\frac{10(\alpha +2)(\alpha +3)(\alpha +4)}{\gamma^{4}}\right\},\\ \; & \left\{\theta_{2,3},\theta_{2,4}\right\}=\\ \; & \;\left\{\frac{3(\alpha +2)}{\gamma^{3}},\frac{15(\alpha +2)(\alpha +4)}{\gamma^{4}}\right\}. \end{array} \end{array}$$

It follows that, in the ultra-relativistic limit, we have

$$\begin{array}{c} \frac{N_{3}}{D_{3}}=\frac{2(\alpha +3)}{\gamma }\;,\;\frac{N_{31}}{D_{3}}=\frac{10}{\gamma }\;,\;C_{5}=\frac{\alpha +4}{\gamma }, \end{array}$$

and

(63) $$\begin{array}{c} \frac{N^{\Pi }}{D_{4}}=-\;\frac{\alpha +4}{\gamma }\;,\;\frac{N^{\Delta }}{D_{4}}=-\;\frac{1}{\alpha +1}\;, \end{array}$$

where the last two equations hold for α≠2 (i.e., a≠2). For a=2, the ultra-relativistic limit of $\frac{N^{\Pi }}{D_{4}}$ and of $\frac{N^{\Delta }}{D_{4}}$ gives the indeterminate form $\left[\frac{0}{0}\right]$. We show (see Appendix B for details) that it can be solved by considering higher order terms for the energy *e*, allowing one to prove that Equation (63) is valid also with a=2, and hence that the closure of the present model is continuous with respect to the parameter α, at the ultra-relativistic limit.

## 7. Principal Subsystems of RET${}_{15}$

For a general hyperbolic system of balance laws, the system with a smaller set of the field equations can be deduced (*principal subsystems*), retaining the property that the convexity of the entropy and the positivity of the entropy production is preserved according to the definition given in [20]. The principal subsystems are obtained by putting some components of the main field as a constant, and the corresponding balance laws are deleted.

Let us recall the system (18). The balance law of $A^{\alpha \beta \gamma }$ is divided into the trace part $A_{\beta }^{\alpha \beta }$ and the traceless part $A^{\alpha <\beta \gamma >}$. As we study below, by deleting the trace part and putting the corresponding component of the main field as zero, we obtain the theory with 14 fields (RET${}_{14}$). On the other hand, by conducting the same procedure on the traceless part, we obtain the theory with 6 fields (RET${}_{6}$). It is remarkable that RET${}_{14}$ and RET${}_{6}$ is the same order in the sense of the principle subsystem, differently from the classical case in which the classical RET${}_{6}$ is a principal subsystem of classical RET${}_{14}$. Moreover, the relativistic Euler theory is deduced as a principal subsystem by deleting the balance laws of $A^{\alpha \beta \gamma }$ and putting the corresponding component of the main field as zero.

### 7.1. RET_14_: 14 Fields Theory

The RET${}_{14}$ is obtained as a principal subsystem of RET${}_{15}$ under the condition $\lambda_{\alpha }^{\alpha }=0$. From (36)${}_{3}$, this condition provides Δ expressed by Π as follows:

(64) $$\begin{array}{cc} \Delta^{\left(14\right)} & =-\frac{c^{2}\alpha_{1}-3\alpha_{2}}{c^{2}\beta_{1}-3\beta_{2}}\Pi =4\frac{N_{a}}{D_{a}}c^{2}\Pi , \end{array}$$

where $N_{a}=D_{4}^{44}+D_{4}^{43}$ and $D_{a}=D_{4}^{34}+D_{4}^{33}$. Then, the independent fields are the following 14 fields: $\left(\rho ,\gamma ,\Pi ,U^{\alpha },q^{\alpha },t^{<\alpha \beta {>}_{3}}\right)$. By deleting the balance equation corresponding to $\lambda_{\alpha }^{\alpha }$, that is, the one of $A_{\beta }^{\alpha \beta }$, the present system of the balance equations is as follows:

(65) $$\begin{array}{c} \partial_{\alpha }V^{\alpha }=0\;,\;\partial_{\alpha }T^{\alpha \beta }=0\;,\;\partial_{\alpha }A^{\alpha <\beta \gamma >}=I^{<\beta \gamma >}. \end{array}$$

With (64), the constitutive equation is modified in this subsystem. For the comparison with the RET${}_{14}$ theory studied in [7], let us denote

$$\begin{array}{c} \frac{N_{1}^{\pi }}{D_{1}^{\pi }}=-\frac{1}{3}\frac{N_{a}}{D_{a}},\;\frac{N_{11}^{\pi }}{D_{1}^{\pi }}=\frac{1}{D_{4}}\left(\frac{N_{a}}{D_{a}}N^{\Delta }+N^{\Pi }\right). \end{array}$$

We can prove the following identity:

$$\begin{array}{c} \frac{N_{b}}{D_{a}}=-\frac{1}{D_{4}}\left(\frac{N_{a}}{D_{a}}N^{\Delta }+N^{\Pi }\right),\;\mathrm{with}\;N_{b}=N^{\Delta 34}+N^{\Delta 33}, \end{array}$$

where $N^{\Delta 33}$ and $N^{\Delta 34}$ are the minor determinants of $N^{\Delta }$, which deletes the third row and third column, and the third row and fourth column, respectively. Then, as a result, instead of (35), the closure for $A^{\alpha \beta \gamma }$ in the present principal subsystem is given by

(66) $$\begin{array}{c} A^{\alpha \beta \gamma }=\left(\rho \;\theta_{0,2}-\frac{3}{c^{2}}\;\frac{N_{1}^{\pi }}{D_{1}^{\pi }}\;\Pi \right)U^{\alpha }U^{\beta }U^{\gamma }\;+\;\left(\rho \;c^{2}\theta_{1,2}-3\;\frac{N_{11}^{\pi }}{D_{1}^{\pi }}\;\Pi \right)U^{(\alpha }h^{\beta \gamma )}\;+\\ +\;\frac{3}{c^{2}}\;\frac{N_{3}}{D_{3}}\;q^{(\alpha }U^{\beta }U^{\gamma )}\;+\;\frac{3}{5}\;\frac{N_{31}}{D_{3}}\;q^{(\alpha }h^{\beta \gamma )}\;+\;3\;C_{5}\;t^{(<\alpha \beta {>}_{3}}U^{\gamma )}\;. \end{array}$$

This result is formally the same as the result of [7] (Equation (56) of the paper). However, there are differences in the coefficients due to the presence of ${\left(mc^{2}+I\right)}^{n}$ instead of $mc^{2}+\;n\;I$ in the integrals.

Similarly, we obtain the production term in this principal subsystem as follows:

(67) $$\begin{array}{c} I^{<\beta \gamma >}=-\;\frac{1}{c^{2}\tau }\;\frac{3N_{1}^{\pi }+N_{11}^{\pi }}{D_{1}^{\pi }}\;\Pi \;U^{<\beta }U^{\gamma >}\;+\;\frac{1}{c^{2}\tau }\left(\frac{\theta_{1,3}}{\theta_{1,2}}\;-\;2\;\frac{N_{3}}{D_{3}}\right)\;q^{(\beta }U^{\gamma )}\;-\;\frac{1}{\tau }\;C_{5}\;t^{<\beta \gamma {>}_{3}}\;. \end{array}$$

This expression (67) is formally the same as the result of [8] (Equation (16) of the paper), except that now we have $\frac{\theta_{1,3}}{\theta_{1,2}}$ instead of $\frac{B_{2}}{B_{4}}$ defined in [8], and the difference of the integral in the coefficients is similar with the case for $A^{\alpha \beta \gamma }.$

*The system* (65) *is symmetric hyperbolic in the main field* $\left(\lambda ,\lambda_{\alpha },\lambda_{<\mu \nu >}\right)$*given respectively by* (36) *with*$\Delta =\Delta^{\left(14\right)}$ *given by* (64).

### 7.2. RET_6_: 6 Fields Theory

We consider the principal subsystem with $\lambda_{<\mu \nu >}=\lambda_{\mu \nu }-\frac{1}{4}\lambda_{\alpha }^{\alpha }g_{\mu \nu }=0$, and then we have

(68) $$\begin{array}{c} \lambda_{\mu \nu }=\frac{1}{4}\lambda_{\alpha }^{\alpha }g_{\mu \nu }. \end{array}$$

By comparing it with (36), we have

$$\begin{array}{c} \left(\alpha_{1}+\frac{\alpha_{2}}{c^{2}}\right)\Pi +\left(\beta_{1}+\frac{\beta_{2}}{c^{2}}\right)\Delta =0,\;q_{\mu }=0,\;t_{<\mu \nu {>}_{3}}=0. \end{array}$$

The first equation indicates that, in this principal subsystem, Δ is expressed with Π as follows:

(69) $$\begin{array}{c} \Delta^{\left(6\right)}=-\frac{c^{2}\alpha_{1}+\alpha_{2}}{c^{2}\beta_{1}+\beta_{2}}\Pi =w\;\Pi \end{array}$$

where

$$w=4c^{2}\frac{D_{4}^{44}-3D_{4}^{43}}{D_{4}^{34}-3D_{4}^{33}}.$$

It should be mentioned that the relation between Δ and Π is different from the case of RET${}_{14}$.

The independent fields are now the 6 fields $\left(\rho ,\gamma ,U^{\alpha },\Pi \right)$, and the balance equations are the following:

(70) $$\begin{array}{c} \partial_{\alpha }V^{\alpha }=0\;,\;\partial_{\alpha }T^{\alpha \beta }=0\;,\;\partial_{\alpha }A_{\;\;\;\;\;\beta }^{\alpha \beta }=I_{\;\;\beta }^{\beta }\;. \end{array}$$

where the energy-momentum tensor is now given, instead of (22), by

(71) $$\begin{array}{c} T^{\alpha \beta }=\frac{e}{c^{2}}\;U^{\alpha }U^{\beta }+\;\left(p\;+\;\Pi \right)h^{\alpha \beta }. \end{array}$$

and, from (35),

(72) $$\begin{array}{c} A_{\;\;\;\;\;\beta }^{\alpha \beta }=\left\{\rho c^{2}\left(\theta_{0,2}-\theta_{1,2}\right)+A_{1}\right\}\Pi U^{\alpha }, \end{array}$$

where

$$\begin{array}{c} A_{1}=-\frac{1}{4c^{2}}\left\{\left(1+3\frac{N^{\Delta }}{D_{4}}\right)\frac{c^{2}\alpha_{1}+\alpha_{2}}{c^{2}\beta_{1}+\beta_{2}}-12c^{2}\frac{N^{\Pi }}{D_{4}}\right\}=\frac{D_{4}^{44}-3D_{4}^{43}+3N^{\Delta 34}-9N^{\Delta 33}}{D_{4}^{34}-3D_{4}^{33}}. \end{array}$$

Similarly, from (45), we obtain

(73) $$\begin{array}{c} I_{\;\;\beta }^{\beta }=-\frac{A_{1}}{\tau }\Pi . \end{array}$$

The corresponding Lagrange multiplier to $A_{\;\;\;\;\;\beta }^{\alpha \beta }$ is $\psi =\frac{1}{4}\lambda_{\alpha }^{\alpha }$, which is obtained from (68) as follows:

(74) $$\begin{array}{c} \psi =c^{2}\frac{\alpha_{1}\beta_{2}-\alpha_{2}\beta_{1}}{c^{2}\beta_{1}+\beta_{2}}\Pi . \end{array}$$

*The system* (70) *with* (71) *and* (72) *is symmetric hyperbolic in the main field* $\left(\lambda ,\lambda_{\alpha },\psi \right)$*given respectively by (see* (36)${}_{1,2}$ *):*

(75) $$\lambda =-\frac{g+c^{2}}{T}+\left(a_{1}+a_{2}\;w\right)\;\Pi ,\;\lambda_{\alpha }=\frac{1}{T}\left[1+(b_{1}+b_{2}\;w)\Pi \right]U_{\alpha },$$

*and ψ given by*(74).

The closed field equations with the material derivative are obtained as follows:

(76) $$\begin{array}{c} \begin{array}{cc} \; & \dot{\rho }+\rho \;\partial_{\alpha }\;U^{\alpha }=0\;,\;\\ \; & \frac{e+p+\Pi }{c^{2}}\;{\dot{U}}_{\delta }\;+\;h_{\delta }^{\mu }\;\partial_{\mu }\left(p+\Pi \right)=0\;,\;\\ \; & \dot{e}\;+\;\left(e+p+\Pi \right)\;\partial_{\alpha }U^{\alpha }=0\;,\\ \; & \dot{\Pi }\;+\;\frac{\rho c^{2}\left(\theta_{0,2}^{\prime }-\theta_{1,2}^{\prime }\right)}{A_{1}}\dot{\gamma }\;+\;\frac{{\dot{A}}_{1}}{A_{1}}\Pi \;+\;\Pi \partial_{\alpha }U^{\alpha }=-\;\frac{\Pi }{\tau }. \end{array} \end{array}$$

Taking into account

(77) $$h_{\delta }^{\mu }\;\partial_{\mu }\left(p+\Pi \right)=U_{\delta }\frac{\dot{p}+\dot{\Pi }}{c^{2}}-\partial_{\delta }\left(p+\Pi \right),$$

and from (12):

(78) $$\dot{e}=c^{2}\left(\dot{\rho }\omega +\rho \omega^{\prime }\dot{\gamma }\right),\;\dot{p}=\frac{c^{2}}{\gamma^{2}}\left(\gamma \dot{\rho }-\rho \dot{\gamma }\right),$$

the system (76) can be put in the normal form:

(79) $$\begin{array}{cc} \; & \dot{\rho }+\rho \;\partial_{\alpha }\;U_{\alpha }=0\;,\;\\ \; & \left(\rho +\frac{\rho \varepsilon +p+\Pi }{c^{2}}\right)\;{\dot{U}}_{\delta }\;-\partial_{\delta }\left(p+\Pi \right)-\frac{\left(p+\Pi \right)}{c^{2}}\left[1-\frac{1}{A_{1}\omega^{\prime }}\left(A_{1}^{\prime }\frac{\Pi }{\rho c^{2}}+\frac{A_{1}}{\gamma^{2}}+\theta_{0,2}^{\prime }-\theta_{1,2}^{\prime }\right)\right]U_{\delta }\;\partial_{\alpha }U^{\alpha }=\frac{\Pi }{\tau c^{2}}U_{\delta }\;,\;\\ \; & \rho c^{2}\omega^{\prime }\;\dot{\gamma }\;+\;\left(p+\Pi \right)\;\partial_{\alpha }U^{\alpha }=0\;,\\ \; & \dot{\Pi }\;+\left\{\Pi -\frac{p+\Pi }{\rho c^{2}A_{1}\omega^{\prime }}\left[A_{1}^{\prime }\Pi +\rho c^{2}\left(\theta_{0,2}^{\prime }-\theta_{1,2}^{\prime }\right)\right]\right\}\partial_{\alpha }U^{\alpha }=-\;\frac{\Pi }{\tau }. \end{array}$$

It is extremely interesting that in the relativistic theory the acceleration is influenced by the relaxation time trough the right hand side of (79)${}_{2}$, and this may be important for the application to the problems of cosmology.

### 7.3. RET_5_: Euler 5 Fields Theory

Let us consider the principal subsystem with $\lambda_{\mu \nu }=0$. This indicates that any nonequilibrium variables are set to be zero, i.e.,

(80) $$\begin{array}{c} \Pi =\Delta =0,\;t_{<\mu \nu {>}_{3}}=0,\;q_{\alpha }=0. \end{array}$$

The independent fields are the 5 fields $\left(n,U^{\alpha },\gamma \right)$, and the balance equations are

(81) $$\begin{array}{c} \partial_{\alpha }V^{\alpha }=0\;,\;\partial_{\alpha }T^{\alpha \beta }=0, \end{array}$$

with

(82) $$\begin{array}{c} T^{\alpha \beta }=\frac{e}{c^{2}}\;U^{\alpha }U^{\beta }+\;ph^{\alpha \beta }. \end{array}$$

*The deduced system is the one of the relativistic Euler theory, and the system (81) becomes symmetric in the main field $\left(\lambda =-\left(g+c^{2}\right)/T,\lambda_{\alpha }=U_{\alpha }/T\right)$, as obtained first by Ruggeri and Strumia in [21].*

## 8. Maxwellian Iteration and Phenomenological Coefficients

In order to find the parabolic limit of a system (47) and to obtain the corresponding Eckart equations, we adopt the Maxwellian iteration [31] on (47), in which only the first order terms with respect to the relaxation time are retained. The phenomenological coefficients, that is, the heat conductivity χ, the shear viscosity μ, and the bulk viscosity ν, are identified with the relaxation time.

The method of the Maxwellian iteration is based on putting to zero the nonequilibrium variables on the left side of Equation (47):

(83) $$\begin{array}{c} \begin{array}{cc} \; & \dot{\rho }-\rho \;h^{\beta \alpha }\;\partial_{\beta }\;U_{\alpha }=0\;,\\ \; & \frac{e+p}{c^{2}}\;h_{\delta \beta }\;{\dot{U}}^{\beta }\;-\;h_{\delta }^{\mu }\;\partial_{\mu }p=0\;,\\ \; & \dot{e}\;-\;\left(e+p\right)\;h_{\alpha }^{\mu }\;\partial_{\mu }U^{\alpha }=0\;,\\ \; & \frac{c^{2}}{3}h_{\delta \beta }\;\left(\dot{\rho }\theta_{1,2}+\rho \theta_{1,2}^{\prime }\dot{\gamma }\right)-\;\frac{1}{3}\rho c^{2}\theta_{1,2}\;\left[h_{\delta \beta }\;h_{\alpha }^{\mu }\;\partial_{\mu }\;U^{\alpha }\;+\;2\;h_{\theta (\delta }\;h_{\beta )}^{\mu }\;\partial_{\mu }\;U^{\theta }\right]=\\ \; & \;=\frac{1}{\tau }\;\left(\frac{1}{4}\;\frac{N^{\Delta }}{D_{4}}\;\frac{\Delta }{c^{2}}\;+\;\frac{N^{\Pi }}{D_{4}}\;\Pi \right)h_{\delta \beta }\;-\;\frac{1}{\tau }\;C_{5}\;t_{<\delta \beta {>}_{3}}\;,\\ \; & h_{\beta \delta }\;{\dot{U}}^{\beta }\left(\rho \theta_{0,2}c^{2}+\frac{2}{3}\rho c^{2}\theta_{1,2}\right)\;-\;h_{\delta }^{\mu }\;\;\frac{c^{4}}{3}\partial_{\mu }\;\left(\rho \theta_{1,2}\right)=\frac{1}{\tau }\;\left(\frac{N_{3}}{D_{3}}\;-\;\frac{\theta_{1,3}}{2\;\theta_{1,2}}\right)\;q_{\delta }\;,\\ \; & c^{4}\left(\dot{\rho }\theta_{0,2}+\rho \theta_{0,2}^{\prime }\dot{\gamma }\right)\;-\;\;\rho c^{4}\left(\theta_{0,2}+\frac{2}{3}\theta_{1,2}\right)h_{\alpha }^{\mu }\;\partial_{\mu }\;U^{\alpha }\;=-\;\frac{1}{4\;\tau }\;\Delta \;. \end{array} \end{array}$$

From the first three equations of (83) and taking into account $p=\rho c^{2}/\gamma ,e=\rho c^{2}\omega \left(\gamma \right)$ (see (12)), we can deduce

(84) $$\begin{array}{c} \dot{\rho }=\rho \;h^{\mu \alpha }\partial_{\mu }\;U_{\alpha },\;h_{\delta }^{\mu }\;\partial_{\mu }\;\rho =\rho \frac{\omega \gamma +1}{c^{2}}\;h_{\delta \beta }U^{\mu }\;\partial_{\mu }\;U^{\beta }\;+\;\frac{\rho }{\gamma }\;h_{\delta }^{\mu }\;\partial_{\mu }\;\gamma ,\;\;\dot{\gamma }=\frac{1}{\gamma \omega^{\prime }}\;h^{\mu \alpha }\partial_{\mu }\;U_{\alpha }\;. \end{array}$$

Putting (84) in the remaining Equation (83)${}_{4,5,6}$, we obtain the solution

(85) $$\begin{array}{c} \begin{array}{cc} \; & q_{\beta }=-\chi \;h_{\beta }^{\alpha }\left[\partial_{\alpha }T-\frac{T}{c^{2}}U^{\mu }\partial_{\mu }U^{\alpha }\right],\\ \; & \Pi =-\nu \;\partial_{\alpha }U^{\alpha },\\ \; & t_{<\beta \delta {>}_{3}}=2\mu \;h_{\beta }^{\alpha }\;h_{\delta }^{\mu }\partial_{<\alpha }U_{\mu >},\\ \; & \Delta =\sigma \;\partial_{\alpha }U^{\alpha }, \end{array} \end{array}$$

with

(86) $$\begin{array}{c} \begin{array}{cc} \; & \chi =-\frac{2\rho c^{2}}{3B^{q}T}\left[3\theta_{0,2}+\theta_{1,2}\left(1-\omega \;\gamma \right)\right],\\ \; & \nu =-\frac{\rho c^{2}}{3B_{2}^{\Pi }}\left\{\frac{2}{3}\theta_{1,2}-\frac{\theta_{1,2}^{\prime }}{\gamma \omega^{\prime }}+3\frac{N^{\Delta }}{D_{4}}\left(\frac{2}{3}\theta_{1,2}-\frac{\theta_{0,2}^{\prime }}{\gamma \omega^{\prime }}\right)\right\},\\ \; & \mu =-\frac{\rho c^{2}}{3B^{t}}\theta_{1,2}\;, \end{array} \end{array}$$

and

$$\sigma =\frac{\rho }{B_{1}^{\Delta }}\left(\frac{2}{3}\theta_{1,2}-\frac{\theta_{0,2}^{\prime }}{\gamma \omega^{\prime }}\right),$$

where $B_{2}^{\Pi },\;B^{q},\;B^{t}$ are explicitly given by (44) with the relaxation time τ.

As the first three equations in (85) are the Eckart equations, we deduce that χ,ν,μ are the heat conductivity, the bulk viscosity, and the shear viscosity, respectively. In addition, we have a new phenomenological coefficient σ, but as Δ doesn’t appear in either $V^{\alpha }$ or $T^{\alpha \beta }$ (see Equation (22) or the first three equations in (47)), we arrive at the conclusion that the present theory converges to the Eckart one formed in the first three block equations of (47) with constitutive Equation (85), in which the heat conductivity, bulk viscosity, and shear viscosity are explicitly given by (86)${}_{1,2,3}$.

We introduce, as in [9], the dimensionless variables, as follows:

(87) $$\begin{array}{c} \begin{array}{cc} \; & \bar{\chi }=\frac{\rho T\chi }{p^{2}\tau }=-\frac{2}{3}\gamma^{2}\frac{3\theta_{0,2}+\theta_{1,2}\left(1-\omega \;\gamma \right)}{\frac{\theta_{1,3}}{\theta_{1,2}}\;-2\frac{N_{3}}{D_{3}}},\\ \; & \bar{\nu }=\frac{\nu }{p\tau }=-\frac{1}{3}\frac{\gamma }{\frac{N^{\Pi }}{D_{4}}}\left\{\frac{2}{3}\theta_{1,2}-\frac{\theta_{1,2}^{\prime }}{\gamma \omega^{\prime }}+3\frac{N^{\Delta }}{D_{4}}\left(\frac{2}{3}\theta_{1,2}-\frac{\theta_{0,2}^{\prime }}{\gamma \omega^{\prime }}\right)\right\},\\ \; & \bar{\mu }=\frac{\mu }{p\tau }=\frac{\gamma }{3C_{5}}\theta_{1,2}\;, \end{array} \end{array}$$

which are functions only of γ.

### 8.1. Ultra-Relativistic and Classical Limit of the Phenomenological Coefficients

Taking into account Equations (62) and (63), it is simple to obtain the limit of (87) when γ→0:

$$\begin{array}{c} \begin{array}{c} {\bar{\chi }}_{\mathrm{ultra}}=0,\;{\bar{\nu }}_{\mathrm{ultra}}=\frac{2}{3}\frac{\alpha^{2}-4}{\left(1+\alpha )(4+\alpha \right)},\;{\bar{\mu }}_{\mathrm{ultra}}=\frac{2+\alpha }{4+\alpha }. \end{array} \end{array}$$

In particular, in the most significant case in which a≤2 for which α=2, we have

(88) $$\begin{array}{c} \begin{array}{c} {\bar{\chi }}_{\mathrm{ultra}}=0,\;{\bar{\nu }}_{\mathrm{ultra}}=0,\;{\bar{\mu }}_{\mathrm{ultra}}=\frac{2}{3}. \end{array} \end{array}$$

Instead, in the classical limit for which γ→∞, it was proved in [7] that the internal energy ε converges to the classical internal energy of polytropic gas: $\varepsilon =\left(D/2\right)\left(k_{B}/m\right)T$. Therefore, from (13), ω converges to

(89) $$\omega_{\mathrm{class}}=1+\frac{D}{2\gamma }.$$

In the present case, using (89), it is not difficult to find $\theta_{h,j}$ deduced in (17) in the limit γ→∞, as follows:

(90) $$\begin{array}{c} \begin{array}{cc} \; & \left\{\theta_{0,0}\;,\theta_{0,1}\;,\theta_{0,2}\;,\theta_{0,3}\;,\theta_{0,4}\right\}=\left\{1\;,1+\frac{D}{2\gamma }\;,1+\frac{D}{\gamma }\;,1+\frac{3D}{2\gamma }\;,1+\frac{2D}{\gamma }\right\},\\ \; & \left\{\theta_{1,1}\;,\theta_{1,2}\;,\theta_{1,3}\;,\theta_{1,4}\right\}=\left\{\frac{1}{\gamma }\;,\frac{3}{\gamma }\;,\frac{6}{\gamma }\;,\frac{10}{\gamma }\right\}\;,\\ \; & \left\{\theta_{2,3}\;,\theta_{2,4}\right\}=\left\{\frac{3}{\gamma^{2}}\;,\frac{15}{\gamma^{2}}\right\}. \end{array} \end{array}$$

Therefore, in the classical limit, we have

(91) $$\begin{array}{c} \frac{N_{3}}{D_{3}}=2\;,\;\frac{N_{31}}{D_{3}}=\frac{10}{2+D}\;,\;C_{5}=1\;,\;\frac{N^{\Pi }}{D_{4}}=-1\;,\;\frac{N^{\Delta }}{D_{4}}=-\frac{2}{D}\;, \end{array}$$

and we find from (87)

(92) $$\begin{array}{c} \begin{array}{c} {\bar{\chi }}_{\mathrm{class}}=\frac{D+2}{2}\;,\;{\bar{\nu }}_{\mathrm{class}}=\frac{2(D-3)}{3D}\;,\;{\bar{\mu }}_{\mathrm{class}}=1\;, \end{array} \end{array}$$

which are in perfect agreement with the phenomenological coefficients of the classical RET theory [2].

### 8.2. Phenomenological Coefficients in RET_14_ and RET_6_

By conducting the Maxwellian iteration to RET${}_{14}$ as a principal subsystem of RET${}_{15}$, we may expect that a different bulk viscosity appears. This is because Δ is related to Π by (64), and it affects the balance laws corresponding to Π in RET${}_{14}$. In fact, from (66) and (67), we can obtain the closed field equations for Π, and then, through the Maxwellian iteration, as has been done in [9], we obtain the bulk viscosity for RET${}_{14}$ as follows:

(93) $$\begin{array}{c} {\bar{\nu }}_{14}=\frac{\frac{1}{\omega^{\prime }}\left(\theta_{0,2}^{\prime }+\frac{1}{3}\theta_{1,2}^{\prime }\right)-\frac{8}{9}\gamma \theta_{1,2}}{\left(-1+\frac{N^{\Delta }}{D_{4}}\right)\frac{N_{a}}{D_{a}}+\frac{N^{\Pi }}{D_{4}}}. \end{array}$$

We remark that the heat conductivity and the shear viscosity is the same between RET${}_{15}$ and RET${}_{14}$.

Similarly, from (79)${}_{4}$, we obtain the bulk viscosity estimated by RET${}_{6}$ as follows:

(94) $$\begin{array}{cc} \; & {\bar{\nu }}_{6}=-\frac{\theta_{0,2}^{\prime }-\theta_{1,2}^{\prime }}{\omega^{\prime }A_{1}}. \end{array}$$

It should be noted that, in the classical case studied in [15], the bulk viscosities of RET${}_{15}$, RET${}_{14}$, and RET${}_{6}$ are the same. In fact, in the classical limit, ${\bar{\nu }}_{14}$ and ${\bar{\nu }}_{6}$ coincide with ${\bar{\nu }}_{\mathrm{class}}$. However, due to the mathematical structure of the relativity (i.e., the scalar fields Π and Δ appear together in the triple tensor), the method of the principal subsystem dictates the difference of the subsystems.

### 8.3. Heat Conductivity, Bulk Viscosity, and Shear Viscosity in Diatomic Gases

Inserting (60), after cumbersome calculations (easy with Mathematica^®^), we can obtain the phenomenological coefficients in the diatomic case:

$$\begin{array}{cc} \; & \bar{\chi }=-\frac{\gamma \left(\gamma^{2}+2\gamma G-8\right){\left\{\gamma^{4}\left(G^{2}-1\right)+2\gamma^{2}\left(G^{2}+2\right)-5\gamma^{3}G-16\gamma G+32\right\}}^{2}}{{\left(\gamma G-4\right)}^{3}\left\{\gamma \left[-\gamma^{5}+5\gamma^{3}+48\gamma +\left(\gamma^{4}-6\gamma^{2}-12\right)\gamma G^{2}+\left(-5\gamma^{4}+12\gamma^{2}+96\right)G\right]-192\right\}},\\ \; & \bar{\mu }=\frac{{\left(\gamma^{2}+2\gamma G-8\right)}^{2}}{\left(\gamma G-4\right)\left\{4\left(\gamma^{2}-8\right)+\gamma \left(\gamma^{2}+8\right)G\right\}},\\ \; & \bar{\nu }=\frac{g_{1}}{3\left(\gamma G-4\right)g_{2}}, \end{array}$$

with

$$\begin{array}{cc} \; & g_{1}=4\gamma^{15}G{\left(G^{2}-1\right)}^{2}+81920\gamma^{3}G\left(7G^{2}+20\right)-196608\gamma^{2}\left(7G^{2}+4\right)+1024\gamma^{5}G\left(21G^{4}+660G^{2}-392\right)-\\ \; & \;4096\gamma^{4}\left(35G^{4}+348G^{2}-56\right)+4\gamma^{14}\left(G^{6}-17G^{4}+21G^{2}-5\right)+\gamma^{13}G\left(7G^{6}-86G^{4}+435G^{2}-256\right)+\\ \; & \;4\gamma^{12}\left(-40G^{6}+193G^{4}-331G^{2}+48\right)+4\gamma^{11}G\left(-14G^{6}+422G^{4}-943G^{2}+500\right)+\\ \; & \;16\gamma^{10}\left(77G^{6}-660G^{4}+677G^{2}-84\right)+16\gamma^{9}G\left(7G^{6}-714G^{4}+2560G^{2}-1108\right)-\\ \; & \;64\gamma^{8}\left(45G^{6}-910G^{4}+1472G^{2}-204\right)+64\gamma^{7}G\left(G^{6}+492G^{4}-2800G^{2}+1760\right)-\\ \; & \;256\gamma^{6}\left(7G^{6}+740G^{4}-1344G^{2}+192\right)+1835008\gamma G-1048576,\\ \; & g_{2}=\gamma^{4}\left(G^{2}-1\right)+\gamma^{2}\left(G^{2}+4\right)-5\gamma^{3}G-8\gamma G+16)[\gamma (2\gamma^{9}G^{2}\left(G^{2}-1\right)+5\gamma^{8}G\left(1-3G^{2}\right)+\\ \; & \;40\gamma^{6}G\left(6-5G^{2}\right)+64\gamma^{4}G\left(11G^{2}-25\right)+512\gamma^{2}G\left(G^{2}+14\right)-1024\gamma \left(3G^{2}+5\right)+\\ \; & \;\gamma^{7}\left(19G^{4}-17G^{2}+28\right)-4\gamma^{5}\left(13G^{4}-198G^{2}+60\right)-32\gamma^{3}\left(G^{4}+108G^{2}-52\right)+8192G)-8192]. \end{array}$$

**Figure 1:**
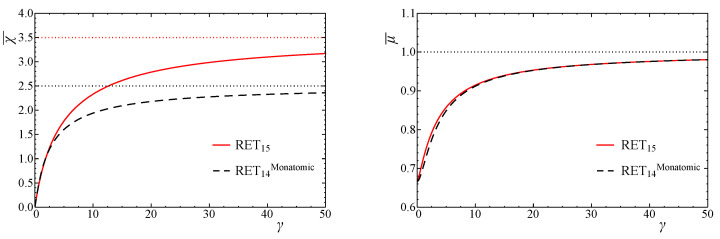
Dependence of $\bar{\chi }$ (**left**) and $\bar{\mu }$ (**right**) for diatomic (red solid line) and monatomic (black dashed line) gases on γ. The dotted line indicates the corresponding value in the classical limit. In the ultra-relativistic limit (γ→0), ${\bar{\chi }}_{\mathrm{ultra}}=0,{\bar{\mu }}_{\mathrm{ultra}}=2/3$ both for monatomic and diatomic gases. In the classical limit (γ→∞), ${\bar{\chi }}_{\mathrm{class}}=2.5,{\bar{\mu }}_{\mathrm{class}}=1$ for monatomic gas, and ${\bar{\chi }}_{\mathrm{class}}=3.5,{\bar{\mu }}_{\mathrm{class}}=1$ for diatomic gas.

**Figure 2:**
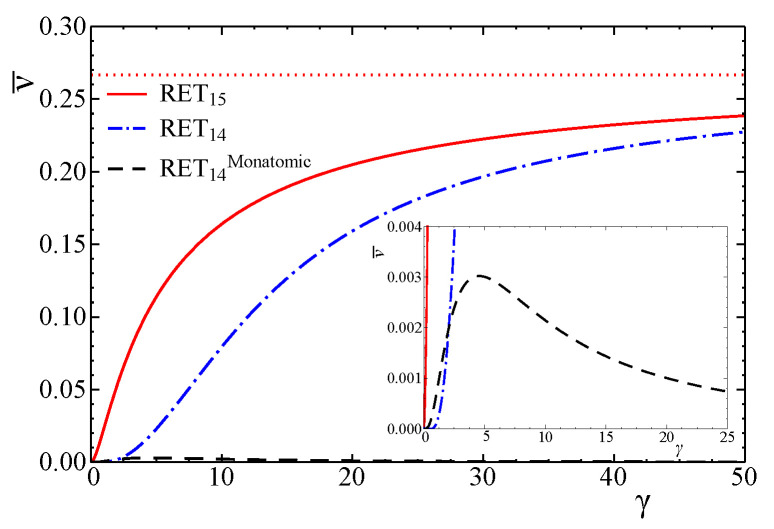
Dependence of $\bar{\nu }$ for diatomic (red solid line) and monatomic (black dashed line) gases on γ. The prediction by RET${}_{14}$ as a principal subsystem of RET${}_{15}$ is also shown with the dotted line. In the ultra-relativistic limit (γ→0), ${\bar{\nu }}_{\mathrm{ultra}}=0$ both for monatomic and diatomic gases. In the classical limit (γ→∞), ${\bar{\nu }}_{\mathrm{class}}=0$ for monatomic gas, and ${\bar{\nu }}_{\mathrm{class}}=4/15$ for diatomic gas.

Let us compare the phenomenological coefficients with the ones for the monatomic case obtained in [9]. In Figure 1, we plot the dependence of the dimensionless heat conductivity and shear viscosity on γ for both diatomic and monatomic cases. Concerning ν, we also plot the dimensionless bulk viscosity of RET${}_{14}$ derived in (93) in Figure 2. We observe that in the ultra-relativistic limit and the classical limit, the figures are in perfect agreement with the limits (88) and (92) (for D=3,5). We remark, as is evidently shown in Figure 2, how small the bulk viscosity in monatomic gas is with respect to that of the diatomic case.

It is also remarkable that the value of the bulk viscosity of RET${}_{6}$ given by (94) is quite near to the one of RET${}_{15}$. For this reason, we omit the plot of ${\bar{\nu }}^{\left(6\right)}$ in the figure. This indicates that RET${}_{6}$ captures the effect of the dynamic pressure in consistency with RET${}_{15}$.

## 9. Classic Limit of the Relativistic Theory

We want to perform the classical limit γ→∞ of the closed relativistic system (47) now. For this purpose, we recall the limits of the coefficients given in (90) and (91). Moreover, taking into account the decomposition $U^{\alpha }\;\equiv \;\left(\Gamma \;c\;,\;v^{i}\right)$, where Γ is the Lorentz factor, we have $\partial_{\alpha }U^{\alpha }=\;\frac{1}{c}\;\partial_{t}\left(\Gamma \;c\right)+\;\partial_{k}\;\left(\Gamma \;v^{k}\right)$, whose limit is $\partial_{i}v^{i}$ because $\partial_{t}\;\Gamma =-\;\Gamma^{3}\frac{v_{i}}{c^{2}}\;\partial_{t}v^{i}$ has zero limit, and a similar evaluation applies to $\partial_{k}\;\Gamma$. Then,

$$\begin{array}{c} \frac{1}{c^{2}}\;U^{\mu }\partial_{\mu }U^{0}=\frac{1}{c^{2}}\;\Gamma \;c\;\frac{1}{c}\;\partial_{t}\;\left(\Gamma \;c\right)\;+\;\frac{1}{c^{2}}\;\Gamma \;v^{k}\;\partial_{k}\;\left(\Gamma \;c\right)\;has\;0\;limit\;,\\ \frac{1}{c^{2}}\;U^{\mu }\partial_{\mu }U^{i}=\frac{1}{c^{2}}\;\Gamma \;c\;\frac{1}{c}\;\partial_{t}\;\left(\Gamma \;v^{i}\right)\;+\;\frac{1}{c^{2}}\;\Gamma \;v^{k}\;\partial_{k}\;\left(\Gamma \;v^{i}\right)\;has\;0\;limit\;. \end{array}$$

Concerning the projection operator in the limit, it is necessary to remember that, with our choice of the metric, $v_{j}=-\;v^{j}$, then

$$\begin{array}{cc} h^{\beta \alpha }= & -\;g^{\beta \alpha }+\frac{U^{\beta }U_{\alpha }}{c^{2}}\;\to \;h^{ij}=-\;g^{ij}+\Gamma^{2}\;\frac{v^{i}v^{j}}{c^{2}}\;\to \;\lim_{c\;\to \;+\infty }\;h^{ij}=-\;g^{ij}=\delta^{ij},\\ \; & \lim_{c\;\to \;+\infty }\;h_{j}^{i}=-\;g_{j}^{i}=-\;diag\;\left(1,\;1,\;1\right)\;. \end{array}$$

While from

$$\begin{array}{c} \begin{array}{cc} \; & 0=U_{\alpha }h^{i\alpha }=\Gamma \;c\;h^{i0}+\Gamma \;v_{k}\;h^{ik}\;\to \;h^{i0}=-\frac{v_{k}}{c}\;h^{ik}\;,\\ \; & \;0=U_{\alpha }h^{0\alpha }=\Gamma \;c\;h^{00}+\Gamma \;v_{k}\;h^{0k}\;\to \;h^{00}=-\frac{v_{k}}{c}\;h^{0k}=-\frac{v_{a}v_{b}}{c^{2}}\;h^{ab}\;. \end{array} \end{array}$$

The last two relations also hold without taking the non-relativistic limit. As a consequence, we have that $\lim_{c\;\to \;+\infty }\;h^{i0}=0$ and $\lim_{c\;\to \;+\infty }\;h^{00}=0$.

The relativistic material derivative (46) of a function *f* converges to the classical material derivative where we continue to indicate it with a dot. Then, the system (47) becomes in the classical limit:

(95) $$\begin{array}{c} \begin{array}{cc} \; & \dot{\rho }+\rho \frac{\partial v_{l}}{\partial x_{l}}=0,\\ \; & \rho {\dot{v}}_{i}+\frac{\partial p}{\partial x_{i}}+\frac{\partial \Pi }{\partial x_{i}}-\frac{\partial \sigma_{\langle ik\rangle }}{\partial x_{k}}=0,\\ \; & \dot{T}+\frac{2T}{Dp}\left\{\left(p+\Pi \right)\frac{\partial v_{l}}{\partial x_{l}}-\sigma_{\langle ik\rangle }\frac{\partial v_{k}}{\partial x_{i}}+\frac{\partial q_{l}}{\partial x_{l}}\right\}=0,\\ \; & \dot{\Pi }+\frac{2}{3}\frac{D-3}{D}p\frac{\partial v_{l}}{\partial x_{l}}+\frac{5D-6}{3D}\Pi \frac{\partial v_{l}}{\partial x_{l}}-\frac{2}{3}\frac{D-3}{D}\sigma_{\langle lk\rangle }\frac{\partial v_{l}}{\partial x_{k}}+\frac{4(D-3)}{3D(D+2)}\frac{\partial q_{l}}{\partial x_{l}}=-\frac{1}{\tau }\;\Pi ,\\ \; & {\dot{\sigma }}_{\langle ij\rangle }+\sigma_{\langle ij\rangle }\frac{\partial v_{l}}{\partial x_{l}}+2\sigma_{\langle l\langle i\rangle }\frac{\partial v_{j\rangle }}{\partial x_{l}}-2\left(p+\Pi \right)\frac{\partial v_{\langle j}}{\partial x_{i\rangle }}-\frac{4}{D+2}\frac{\partial q_{\langle i}}{\partial x_{j\rangle }}=-\frac{1}{\tau }\sigma_{\langle ij\rangle },\\ \; & {\dot{q}}_{i}+\frac{D+4}{D+2}q_{i}\frac{\partial v_{l}}{\partial x_{l}}+\frac{D+4}{D+2}q_{l}\frac{\partial v_{i}}{\partial x_{l}}+\frac{2}{D+2}q_{l}\frac{\partial v_{l}}{\partial x_{i}}\\ \; & \;+\frac{D+2}{2}\frac{p}{\rho T}\left\{\left(p+\Pi \right)\delta_{il}-\sigma_{\langle il\rangle }\right\}\frac{\partial T}{\partial x_{l}}-\frac{p}{\rho^{2}}\left(\Pi \delta_{il}-\sigma_{\langle il\rangle }\right)\frac{\partial \rho }{\partial x_{l}}\\ \; & \;+\frac{1}{\rho }\left\{\left(p-\Pi \right)\delta_{il}+\sigma_{\langle il\rangle }\right\}\left(\frac{\partial \Pi }{\partial x_{l}}-\frac{\partial \sigma_{\langle rl\rangle }}{\partial x_{r}}\right)+\frac{1}{2D}\frac{\partial \Delta }{\partial x_{i}}=-\frac{1}{\tau }\;q_{i},\\ \; & \dot{\Delta }+\left(\frac{D+4}{D}\Delta +8\frac{p}{\rho }\Pi \right)\frac{\partial v_{l}}{\partial x_{l}}-8\frac{p}{\rho }\sigma_{\langle ik\rangle }\frac{\partial v_{i}}{\partial x_{k}}-\frac{8}{\rho }q_{i}\frac{\partial p}{\partial x_{i}}\\ \; & \;+4\left(D+4\right)\frac{p}{\rho T}q_{l}\frac{\partial T}{\partial x_{l}}+\frac{8p}{\rho }\frac{\partial q_{l}}{\partial x_{l}}-\frac{8}{\rho }q_{i}\frac{\partial \Pi }{\partial x_{i}}+\frac{8}{\rho }q_{i}\frac{\partial \sigma_{\langle il\rangle }}{\partial x_{l}}=-\frac{1}{\tau }\Delta , \end{array} \end{array}$$

where $\sigma_{\langle ij\rangle }=-t_{\langle ij\rangle }$. The system (95) coincides perfectly with the classical one obtained recently in [15].

We remark that, as has been studied in [15], for classical polytropic gases, RET${}_{14}$ is derived as a principal subsystem of RET${}_{15}$ by setting Δ=0. Moreover, RET${}_{6}$ is derived from RET${}_{14}$ as a principal subsystem of RET${}_{14}$ by setting $\sigma_{\langle ij\rangle }=0$ and $q_{i}=0$. This corresponds to the fact that, in the classical limit, both $\Delta^{\left(14\right)}$ defined in (64) and $\Delta^{\left(6\right)}$ defined in (69) become zero.

## Data Availability

Not applicable.

## Appendix A. Entropy-Entropy Flux Density

In order to evaluate the entropy density from Equation (20), we need the expression of flnf up to the second order with respect to the nonequilibrium variables. The expansion of the distribution function around an equilibrium state is

$$\begin{array}{c} \begin{array}{cc} \; & f=f_{E}\;\;e^{\frac{-1}{k_{B}}\;\tilde{\chi }}=f_{E}\;\left[1\;-\;\frac{1}{k_{B}}\;\tilde{\chi }\;+\;\frac{1}{2\;k_{B}^{2}}\;{\left(\tilde{\chi }\right)}^{2}\;+\;{\left(\tilde{\chi }\right)}^{3}\left(\cdots \right)\right]\;,\\ \; & with\;\tilde{\chi }={\left(\tilde{\chi }\right)}^{\left(1\right)}+{\left(\tilde{\chi }\right)}^{\left(2\right)}+{\left(\tilde{\chi }\right)}^{\left(3\right)}\;\left(\cdots \right)\;, \end{array} \end{array}$$

defined in (25)${}_{2}$, and the notation $\eta^{\left(i\right)}$ represents the homogeneous part of the generic quantity η at the order *i* with respect to the nonequilibrium variables. With this notation, the quantities ${\left(\lambda -\lambda^{E}\right)}^{\left(1\right)}$, ${\left(\lambda_{\beta }-\lambda_{\beta }^{E}\right)}^{\left(1\right)}$, ${\left(\lambda_{\beta \gamma }\right)}^{\left(1\right)}$ are those of Equation (36).

By composing the above expressions, we see that the distribution function up to the second order is

$$\begin{array}{c} f=f_{E}\;\left\{1\;-\;\frac{1}{k_{B}}\;\left[{\left(\tilde{\chi }\right)}^{\left(1\right)}+{\left(\tilde{\chi }\right)}^{\left(2\right)}\right]+\;\frac{1}{2\;k_{B}^{2}}\;{\left[{\left(\tilde{\chi }\right)}^{\left(1\right)}\right]}^{2}\right\}\;. \end{array}$$

and

$$\begin{array}{c} \begin{array}{cc} f\;\ln f= & f\;\left(-1-\;\frac{1}{k_{B}}\;\tilde{\chi }\right)=f_{E}\;\left\{1\;-\;\frac{1}{k_{B}}\;\left[{\left(\tilde{\chi }\right)}^{\left(1\right)}+{\left(\tilde{\chi }\right)}^{\left(2\right)}\right]+\;\frac{1}{2\;k_{B}^{2}}\;{\left[{\left(\tilde{\chi }\right)}^{\left(1\right)}\right]}^{2}+\cdots \right\}\cdot \\ \; & \;\left\{-1-\;\frac{1}{k_{B}}\;\chi_{E}\;-\;\frac{1}{k_{B}}\;\left[{\left(\tilde{\chi }\right)}^{\left(1\right)}+{\left(\tilde{\chi }\right)}^{\left(2\right)}+\cdots \right]\right\}\\ = & f_{E}\;\ln\;f_{E}\;+\;\frac{1}{k_{B}^{2}}\;f_{E}\;\chi_{E}\;{\left(\tilde{\chi }\right)}^{\left(1\right)}\;+\;\frac{1}{k_{B}^{2}}\;f_{E}\;\chi_{E}\;\left\{{\left(\tilde{\chi }\right)}^{\left(2\right)}\;-\;\frac{1}{2\;k_{B}}\;{\left[{\left(\tilde{\chi }\right)}^{\left(1\right)}\right]}^{2}\right\}+\\ \; & +\;\frac{1}{2\;k_{B}^{2}}\;f_{E}\;{\left[{\left(\tilde{\chi }\right)}^{\left(1\right)}\right]}^{2}\;. \end{array} \end{array}$$

It follows that

$$\begin{array}{c} h^{\alpha }=-k_{B}\;c\int_{R^{3}}\int_{0}^{+\infty }p^{\alpha }f\;\ln\;f\;\varphi \left(I\right)\;d\;I\;d\;P=h_{E}^{\alpha }\;+\;h_{\left(1\right)}^{\alpha }\;+\;h_{\left(2\right)}^{\alpha }\;, \end{array}$$

where

$$\begin{array}{c} \begin{array}{cc} \; & h_{\left(1\right)}^{\alpha }=-\frac{c}{k_{B}}\int_{R^{3}}\int_{0}^{+\infty }p^{\alpha }f_{E}\;\chi_{E}\;{\left(\tilde{\chi }\right)}^{\left(1\right)}\;\varphi \left(I\right)\;d\;I\;d\;P=\\ \; & =-\frac{c}{k_{B}}\int_{R^{3}}\int_{0}^{+\infty }p^{\alpha }f_{E}\;\left[m\;\lambda_{E}+\left(1+\frac{I}{m\;c^{2}}\right)\;\frac{U_{\mu }}{T}\;p^{\mu }\right]\;{\left(\tilde{\chi }\right)}^{\left(1\right)}\;\varphi \left(I\right)\;d\;I\;d\;P, \end{array} \end{array}$$

$$\begin{array}{c} \begin{array}{cc} \; & h_{\left(2\right)}^{\alpha }=-\frac{c}{k_{B}}\int_{R^{3}}\int_{0}^{+\infty }p^{\alpha }f_{E}\;\chi_{E}\;\left\{{\left(\tilde{\chi }\right)}^{\left(2\right)}\;-\;\frac{1}{2\;k_{B}}\;{\left[{\left(\tilde{\chi }\right)}^{\left(1\right)}\right]}^{2}\right\}\;\varphi \left(I\right)\;d\;I\;d\;P\;-\\ \; & \;\;\;\frac{c}{2\;k_{B}}\int_{R^{3}}\int_{0}^{+\infty }p^{\alpha }f_{E}\;{\left[{\left(\tilde{\chi }\right)}^{\left(1\right)}\right]}^{2}\;\varphi \left(I\right)\;d\;I\;d\;P. \end{array} \end{array}$$

Moreover, we have that the moments appearing in system (18) up to the second order are as follows:

$$\begin{array}{c} \begin{array}{cc} \; & \underline{V^{\alpha }=V_{E}^{\alpha }}-\;\frac{m\;c}{k_{B}}\int_{R^{3}}\int_{0}^{+\infty }p^{\alpha }f_{E}\left\{\underline{{\left(\tilde{\chi }\right)}^{\left(1\right)}}+{\left(\tilde{\chi }\right)}^{\left(2\right)}-\;\frac{1}{2\;k_{B}}\;{\left[{\left(\tilde{\chi }\right)}^{\left(1\right)}\right]}^{2}\right\}\varphi \left(I\right)\;d\;I\;d\;P\;,\\ \; & \underline{T^{\alpha \beta }=T_{E}^{\alpha \beta }}-\;\frac{c}{k_{B}}\int_{R^{3}}\int_{0}^{+\infty }p^{\alpha }p^{\beta }\left(1+\frac{I}{m\;c^{2}}\right)f_{E}\left\{\underline{{\left(\tilde{\chi }\right)}^{\left(1\right)}}+{\left(\tilde{\chi }\right)}^{\left(2\right)}-\;\frac{1}{2\;k_{B}}\;{\left[{\left(\tilde{\chi }\right)}^{\left(1\right)}\right]}^{2}\right\}\varphi \left(I\right)\;d\;I\;d\;P\;,\\ \; & \underline{\frac{U_{\alpha }U_{\beta }U_{\gamma }}{c^{4}}\;A^{\alpha \beta \gamma }=\frac{U_{\alpha }U_{\beta }U_{\gamma }}{c^{4}}\;A_{E}^{\alpha \beta \gamma }}-\\ \; & \frac{U_{\alpha }U_{\beta }U_{\gamma }}{c^{4}}\;\frac{c}{m\;k_{B}}\int_{R^{3}}\int_{0}^{+\infty }p^{\alpha }p^{\beta }p^{\gamma }{\left(1+\frac{I}{m\;c^{2}}\right)}^{2}f_{E}\left\{\underline{{\left(\tilde{\chi }\right)}^{\left(1\right)}}+{\left(\tilde{\chi }\right)}^{\left(2\right)}-\;\frac{1}{2\;k_{B}}\;{\left[{\left(\tilde{\chi }\right)}^{\left(1\right)}\right]}^{2}\right\}\varphi \left(I\right)\;d\;I\;d\;P\;. \end{array} \end{array}$$

The underlined terms give 0 for Equation (36), and there remain

$$\begin{array}{c} \begin{array}{cc} \; & -\;\frac{m\;c}{k_{B}}\int_{R^{3}}\int_{0}^{+\infty }p^{\alpha }f_{E}\left\{{\left(\tilde{\chi }\right)}^{\left(2\right)}-\;\frac{1}{2\;k_{B}}\;{\left[{\left(\tilde{\chi }\right)}^{\left(1\right)}\right]}^{2}\right\}\varphi \left(I\right)\;d\;I\;d\;P=0\;,\\ \; & -\;\frac{c}{k_{B}}\int_{R^{3}}\int_{0}^{+\infty }p^{\alpha }p^{\beta }\left(1+\frac{I}{m\;c^{2}}\right)f_{E}\left\{{\left(\tilde{\chi }\right)}^{\left(2\right)}-\;\frac{1}{2\;k_{B}}\;{\left[{\left(\tilde{\chi }\right)}^{\left(1\right)}\right]}^{2}\right\}\varphi \left(I\right)\;d\;I\;d\;P=0\;,\\ \; & \frac{U_{\alpha }U_{\beta }U_{\gamma }}{c^{4}}\;\frac{c}{m\;k_{B}}\int_{R^{3}}\int_{0}^{+\infty }p^{\alpha }p^{\beta }p^{\gamma }{\left(1+\frac{I}{m\;c^{2}}\right)}^{2}f_{E}\left\{{\left(\tilde{\chi }\right)}^{\left(2\right)}-\;\frac{1}{2\;k_{B}}\;{\left[{\left(\tilde{\chi }\right)}^{\left(1\right)}\right]}^{2}\right\}\varphi \left(I\right)\;d\;I\;d\;P=0\;. \end{array} \end{array}$$

The first two of these allow one to prove Equation (52)${}_{1}$ and to write

$$\begin{array}{c} \begin{array}{cc} \; & h_{\left(2\right)}^{\alpha }=-\;\frac{c}{2\;k_{B}}\int_{R^{3}}\int_{0}^{+\infty }p^{\alpha }f_{E}\;{\left[{\left(\tilde{\chi }\right)}^{\left(1\right)}\right]}^{2}\;\varphi \left(I\right)\;d\;I\;d\;P\;. \end{array} \end{array}$$

It is sufficient to substitute the expression of $\tilde{\chi }$ to obtain Equation (52)${}_{2}$.

## Appendix B. Continuity of the Ultra Relativistic Limit for a=2

From (12)${}_{2}$, and by using the recurrence relations (11) and (10), we have

$$\begin{array}{c} \frac{\gamma e}{n\;m\;c^{2}}=\frac{\gamma \;\int_{0}^{+\infty }J_{2,2}^{*}\;\left(1+\frac{I}{mc^{2}}\right)\;\phi \left(I\right)\;d\;I}{\int_{0}^{+\infty }J_{2,1}^{*}\;\phi \left(I\right)\;d\;I}=3+\frac{\int_{0}^{+\infty }J_{0,1}^{*}\;\phi \left(I\right)\;d\;I}{\int_{0}^{+\infty }J_{2,1}^{*}\;\phi \left(I\right)\;d\;I}. \end{array}$$

By introducing the Ruggeri’s numbers $R_{k}$ and using Equations (32)${}_{1}$ of [30], we have

$$\begin{array}{c} \frac{\gamma e}{n\;m\;c^{2}}=3+\frac{3}{-\;\ln\;\gamma }\;R_{-4}=3-\frac{1}{\ln\gamma } \end{array}$$

or

(A1) $$\begin{array}{c} e=\frac{n\;m\;c^{2}}{\gamma }\left(3-\;\frac{1}{\ln\;\gamma }\right)\;. \end{array}$$

Therefore, we have to calculate $D_{4}$, $N^{\Pi }$ and $N^{\Delta }$ with (A1) instead of (61).

In particular, for $D_{4}$ we can add to its fourth line the second one pre-multiplied by $-\frac{1}{3}$, so that it becomes

$$\begin{array}{c} \frac{1}{\gamma \;\ln\gamma }\left(\frac{1}{3}\;,\frac{4}{3\;\gamma }\;,\frac{20}{3\;\gamma^{2}}\;,\frac{4}{3}\;\frac{c^{2}}{\gamma^{2}}\right)\;. \end{array}$$

It follows that, afte r cumbersome calculations that we do not report here for brevity, we have

$$\begin{array}{c} \lim_{\gamma \;\to \;0}\gamma^{9}\;\ln\;\gamma \;D_{4}=\left|\begin{array}{ccccc} 1 & 3 & 12 & 4\\ \; & \; & & \; & \\ 3 & 12 & 60 & 20\\ \; & \; & & \; & \\ 12 & 60 & 360 & 120\\ \; & \; & & \; & \\ \frac{1}{3} & \frac{4}{3} & \frac{20}{3} & \frac{4}{3} \end{array}\right|=-64\;. \end{array}$$

Similarly, for $N^{\Pi }$ we can add to its fourth line the third one multiplied by $-\frac{1}{3}$, so that it becomes

$$\begin{array}{c} \frac{1}{\gamma^{2}\;\ln\gamma }\left(\frac{4}{3}\;,\frac{20}{3\;\gamma }\;,\frac{40}{\gamma^{2}}\;,\frac{8\;c^{2}}{\gamma^{2}}\right)\;. \end{array}$$

It follows that

$$\begin{array}{c} \lim_{\gamma \;\to \;0}\gamma^{10}\;\ln\;\gamma \;N^{\Pi }=-\;\left|\begin{array}{ccccc} 1 & 3 & 12 & 4\\ \; & \; & & \; & \\ 3 & 12 & 60 & 20\\ \; & \; & & \; & \\ 12 & 60 & 360 & 120\\ \; & \; & & \; & \\ \frac{4}{3} & \frac{20}{3} & 40 & 8 \end{array}\right|=384\;. \end{array}$$

Finally, for $N^{\Delta }$ we can add to its third line the second one multiplied by $-\frac{1}{3}$, so that its third line becomes

$$\begin{array}{c} \frac{1}{\gamma \;\ln\gamma }\left(\frac{1}{3}\;,\frac{4}{3\;\gamma }\;,\frac{20}{3\;\gamma^{2}}\;,\frac{4}{3}\frac{c^{2}}{\gamma^{2}}\right)\;. \end{array}$$

It follows that

$$\begin{array}{c} \lim_{\gamma \;\to \;0}\gamma^{9}\;\ln\;\gamma \;N^{\Delta }=\left|\begin{array}{ccccc} 1 & 3 & 12 & 4\\ \; & \; & & \; & \\ 3 & 12 & 60 & 20\\ \; & \; & & \; & \\ \frac{1}{3} & \frac{4}{3} & \frac{20}{3} & \frac{4}{3}\\ \; & \; & & \; & \\ 4 & 20 & 120 & 40 \end{array}\right|=\frac{64}{3}\;. \end{array}$$

By joining all these results we obtain

$$\begin{array}{c} \lim_{\gamma \;\to \;0}\frac{\gamma \;N^{\Pi }}{D_{4}}=-6\;,\;\lim_{\gamma \;\to \;0}\frac{N^{\Delta }}{D_{4}}=-\;\frac{1}{3}\;, \end{array}$$

which confirms (63) also for a=2.
